# Calibration of commercial fisheries echo sounders using seabed backscatter for the estimation of fishery resources

**DOI:** 10.1371/journal.pone.0301689

**Published:** 2024-05-10

**Authors:** Yanhui Zhu, Minami Kenji, Tsutomu Tokeshi, Yoshihiro Nishiyama, Akinori Kasai, Mitsuhiro Matsuura, Hikari Horie, Kazushi Miyashita

**Affiliations:** 1 Field Science Center for Northern Biosphere, Hokkaido University, Hakodate, Japan; 2 Faculty of Marine Science and Technology, Fukui Prefectural University, Obama, Japan; 3 Marine Electronic Products Division, Furuno Electric Co., Ltd., Nishinomiya, Japan; 4 Agriculture and Fisheries Department, Miyazaki Prefectural Advanced Fisheries Training Institute, Miyazaki Prefectural Government, Miyazaki, Japan; 5 Agriculture and Fisheries Department, Fisheries Administration Division, Miyazaki Prefectural Government, Miyazaki, Japan; MARE – Marine and Environmental Sciences Centre, PORTUGAL

## Abstract

Acoustic methods are often used for fisheries resource surveys to investigate fish stocks in a wide area. Commercial fisheries echo sounders, which are installed on most small fishing vessels, are used to record a large amount of data during fishing trips. Therefore, it can be used to collect the basic information necessary for stock assessment for a wide area and frequently. To carry out the quantification for the fisheries echo sounder, we devised a simple method using the backscattering strength of the seabed to perform calibration periodically and easily. In this study, seabed secondary reflections were used instead of primary reflection because the fisheries echo sounders were not equipped with a time-varied gain (TVG) function, and the primary backscattering strength of the seabed was saturated. It was also necessary to use standard values of seabed backscattering strength averaged over a certain area for calibration to eliminate some of the effects of differences in seabed sediment and vessel motions. By using standard values of the seabed secondary reflections, the fisheries echo sounder was calibrated accurately. Our study can provide a reliable framework to calibrate commercial fisheries echo sounders, to improve the estimation and management of fishery resources.

## Introduction

According to the United Nations Convention on the Law of the Sea, the primary management objective of marine fisheries resources is not to deplete resources by fishing and to implement effective management measures to maintain or restore the resources to levels above the maximum sustainable yield (MSY) [1]. However, half of Japan’s fishery stocks are below the MSY level, and about two-thirds are being over-exploited [2]. One of the reasons why most of Japan’s fishery resources are being depleted is that it is difficult to estimate fishery stocks and resource management carried out based on fishery information is inefficient [3]. Therefore, focusing on fisheries-independent surveys and the appropriate management of fishery resources based on scientific results is necessary.

In Japan, acoustic methods are often used in fisheries resource surveys, as they can efficiently estimate biomass over a wide area. Most currently applied acoustic methods employ quantitative echo sounders on board government and research vessels [4]. Although quantitative echo sounders can estimate the backscattering strength of an object, they have several disadvantages, e.g., the echo sounders have limited survey coverage because they are generally installed on large vessels and have limited survey numbers because they require specialized knowledge [5]. Due to these reasons, there has not been much progress in continuous and wide-area fisheries resource surveys using acoustic methods [5]. Therefore, there is a strong need to improve data acquisition methods to ensure that the necessary basic information can be collected over a wide area and frequently for the appropriate assessment of fishery resources.

In this study, we considered using commercial fisheries echo sounders (hereinafter referred to as ‘general echo sounders’) installed on most fishing vessels, instead of quantitative echo sounders to survey fisheries resources. Notably, if general echo sounders could be used like ‘quasi’ quantitative echo sounders, resource surveys could be conducted using fishing vessels, which would greatly advance the evaluation and management of fishery resources and avoid the cost of equipping new vessels with quantitative echo sounders. To attribute quantifiability to general echo sounders, it is important to compensate for the strength of the reflected sound. Therefore, it is essential to calibrate the transducer system that is processing the sound wave [6]. In addition, it is important to periodically check the accuracy of the transducer system, because it is used for long periods in a constantly rough environment. Therefore, to conduct resource surveys using general echo sounders, it is necessary to establish a simple calibration method that allows for the periodic calibrations of transducer systems.

Generally, the calibration of echo sounders is performed using a calibration ball, a standard target with known scattering strength [6]. However, this method is a time- and manpower-intensive calibration method because the calibration ball must be placed on the sound axis of the transducer and worked onboard [7]. For these reasons, carrying out periodic calibrations on fishing vessels takes much work. Therefore, in this study, we considered a direct calibration method using the seabed, which does not change significantly temporally or physically, instead of a calibration ball. Calibration methods using seabed backscattering strength have only been validated with scientific echo sounders such as split-beam and multi-beam and only primary reflections on the seabed were used [8–11]. Since there is little verification with general echo sounders, which are single beams, it is necessary to examine the validity of the calibration method using the seabed with general echo sounders. In addition, it is also important to consider not only primary reflections on the seabed but also secondary reflections.

The standard value for calibration using the seabed is the backscattering strength in a given area measured with a calibrated general echo sounder. When the fishing vessel has the opportunity to pass over this area where a standard value has been established, the backscattering strength of the seabed can be measured in the same manner. If it is almost equal to the standard value, the transducer system of the general echo sounder can be calibrated, with no anomalies. Thus, measuring the backscattering strength of the seabed allows for the easy calibration of the transducer system. From the above, in this study, we aim to demonstrate that the acoustic backscattering strength of the seabed can be used to calibrate general echo sounders directly.

## Methods

### Mechanisms of general echo sounders

**Fig 1:**
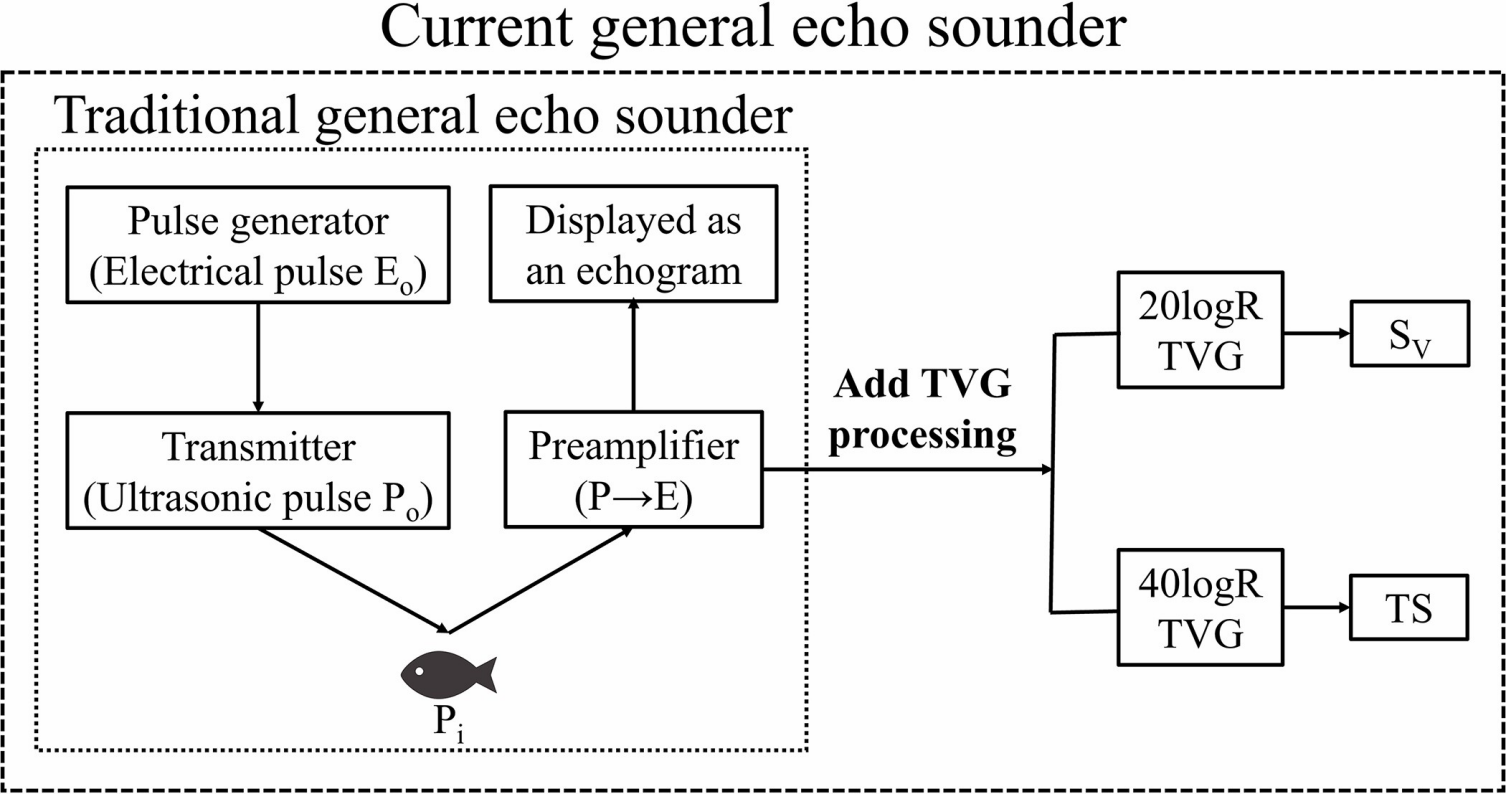
The imagery of general echo sounder evolution. Traditional general echo sounders could only capture fish signals. However, current general echo sounders can calculate backscattering strength by adding the TVG function to those fish signals. R is the range, S_V_ is the volume backscattering strength for multiple targets, and TS is the target strength for a single target.

Conventional general echo sounders are generally used to detect fish schools. However, in recent years, the time-varied gain (TVG) function has been incorporated into the received echoes of general echo sounders (Fig 1). Therefore, the backscattering strength of the object targets can be calculated from the echoes obtained by a general echo sounder. Note that the TVG function is to compensate for the range dependence of the echo [12–14]. When echoes propagate through seawater, they undergo spherical spreading and absorption attenuation. Spherical spreading is the attenuation due to spherical diffusion, with the characteristic of R^-2^ for the range R (m). Absorption attenuation is the attenuation due to energy absorption by seawater’s components. It is exponential with the 10^(-αR/10)^ characteristic for the absorption attenuation coefficient α (dB/m). Unlike the general echo sounder, the TVG function is included in the main unit of the quantitative echo sounder. Therefore, the echoes obtained from the quantitative echo sounder are the values after the TVG calculation. This is the characteristic difference between quantitative and general echo sounders.

In this study, we used the general echo sounders manufactured by FURUNO, which did not have a function to automatically calculate the received echo level of the target. The output data of the general echo sounders were the log conversion data (with the digit values being 0–255), proportional to the reception voltage level. The following equation was used to convert the log conversion data into the received echo level (EL, dBμV).

EL=digit×(a+a’), (1)

where a is the specific coefficient of each transducer at the time of log conversion, and a’ is a specific coefficient that includes each transducer’s frequency and temperature variation characteristics. By adding the distance attenuation level, acquired from the TVG calculation to the received echo level, we accurately calculated the acoustic backscattering strength of the targets. Since the TVG function differs for single and multiple targets [6], 40logR was used for single-target calculations and 20logR for multiple-target calculations in this study.

Target strength TS (dB re 1 m^2^, hereinafter referred to as dB) is a logarithmic measure of the ts, which is the proportion of the incident energy that is backscattered by a single target. The ts is normally described using the backscattering cross-section σ_bs_ (m^2^) and distance R (m) from the target [15–17]. The scattered sound pressure wave P_ts_ (Pa) from the target returns to the transducer under the influence of two types of attenuation and beam pattern. Then, the transducer converts scattered sound signals into electrical signals and sends them to the preamplifier. In the preamplifier, the receiver sensitivity M (V/Pa) and the gain of the front-end amplifier G_R_ are used to output the voltage E_ts_ (V). The E_ts_ from a single target at propagation distance R can be as

$${E_{ts}}^{2}={\left(P_{o}\;M\;G_{R}\right)}^{2}R^{‐4}\;10^{\left(‐2\alpha R/10\right)}b^{4}\;\sigma_{bs}/R^{2}.$$ (2)

Where P_o_ (Pa) is the incident transmission sound pressure amplitude at the reference range from the transducer front, b is the beam pattern (function of direction θ describing the amplitude sensitivity). The ‘P_o_MG_R_’ is called the factor of transmit and receive (denoted by K_TR_), which depends on the transducer used and is determined by calibration [7,18]. The TVG calculations of the distance attenuation items included in Eq (2) yield a logarithmic distance characteristic of 40logR + 2αR [13,19]. Using this distance characteristic to decibel-converted Eq (2), we calculated the TS using the following equation.

TS=EL−KTR+40logR+2αR, (3)

where TS is 10 log σ_bs_/R^2^, EL is 10 log E_ts_, and KTR is 10 log K_TR_. This equation is the basic general echo sounder equation to calculate the echo of an individual target [20].

　When calculating the volume backscatter strength S_V_ (dB re 1 m^-1^, hereinafter referred to as dB) of the group echoes, as there were multiple targets, the voltage E_sv_ (V) was attributed to the group echoes synthesized into the backscattering volume V (m^3^) for the distance R [21], which can be expressed as follows,

$${E_{sv}}^{2}={\left(P_{o}\;M\;G_{R}\right)}^{2}R^{‐4}\;10^{\left(‐2\alpha R/10\right)}V\;s_{v}.$$ (4)

Where s_v_ (m^-1^) is the volume backscattering coefficient. The backscattering volume V (m^3^), which is the shell thickness multiplied by the effective cross-sectional area of the beam, was calculated from the volume element of thickness cτ/2 [22], using the pulse width, τ, sound speed, c (m/s), and a two-way equivalent beam solid angle of a transducer, Ψ as

$$V=\Psi \;R^{2}\;c\tau /2.$$ (5)

Substitute Eq (5) into Eq (4), a logarithmic distance characteristic of 20logR + 2αR was obtained [10,23]. Using this 20logR TVG correction, we calculated the S_V_ using the following equation,

$$S_{V}=EL-KTR+20\log R+2\alpha R-10\;\log(\Psi \;c\tau /2).$$ (6)

Where S_V_ is 10 log s_v_, EL is 10 log E_sv_.

Additionally, as s_v_ was proportional to the distribution density per unit volume, the final value used in the resource calculation was considered as the distribution density per unit area backscatter coefficient (s_a_, m^2^/m^2^). This quantity is a measure of the energy returned from a layer between two ranges and defined as the integral of s_v_ concerning depth through the layer [24]. It can be expressed by the following equation,

$$s_{a}=\int_{R1}^{R2}dR\;s_{v}=n\;ts.$$ (7)

Where R_1_ and R_2_ represent the lower and upper ranges of the volume over which s_v_ is being integrated. Dividing the calculated s_a_ value by the ts, the number of the target species ‘n’ is calculated, and the distribution density per area within a specific sea area is also obtained [25]. The log measure of s_a_, area backscattering strength S_a_ (dB re 1 m^2^/m^2^, hereinafter referred to as dB), is also often used to estimate fish abundance.

### Survey area and investigation

In this study, the survey was conducted in the coastal waters of Shimaura Island, Miyazaki, Japan, from 3 July 2020 to 6 July 2020, using the medium-sized purse seiner vessel Kakutomaru. In addition, we decided to use a quantitative echo sounder at the same time to verify the measurement results of the general echo sounder. We used a general echo sounder (FCV-1500L, 15/200 kHz) manufactured by Furuno along with a quantitative echo sounder (KSE300, 38/120 kHz) manufactured by Sonic (Table 1). Note that the general echo sounder, which is a single beam, was set to a strong transmit power even at high frequency to capture fish school responses in deeper waters. In the case of the general echo sounder, which is a split beam, the transmit power at a higher frequency was lower than at a lower frequency to avoid nonlinear effects.

**Table 1:** Settings for the general and quantitative echo sounders used in this study.

	Specification			
	General echo sounder		Quantitative echo sounder	
Transducer	FCV-1500L		T-178	T-182
Frequency (kHz)	15	200	38	120
-3 dB beam width (°)	32	6	8.5	8.5
Transmit power (kw)	1	2	3	1.5
Beam type	Single		Split	
Pulse width (ms)	0.6		0.6	
Ping rate (s)	1.4		0.2	

**Fig 2:**
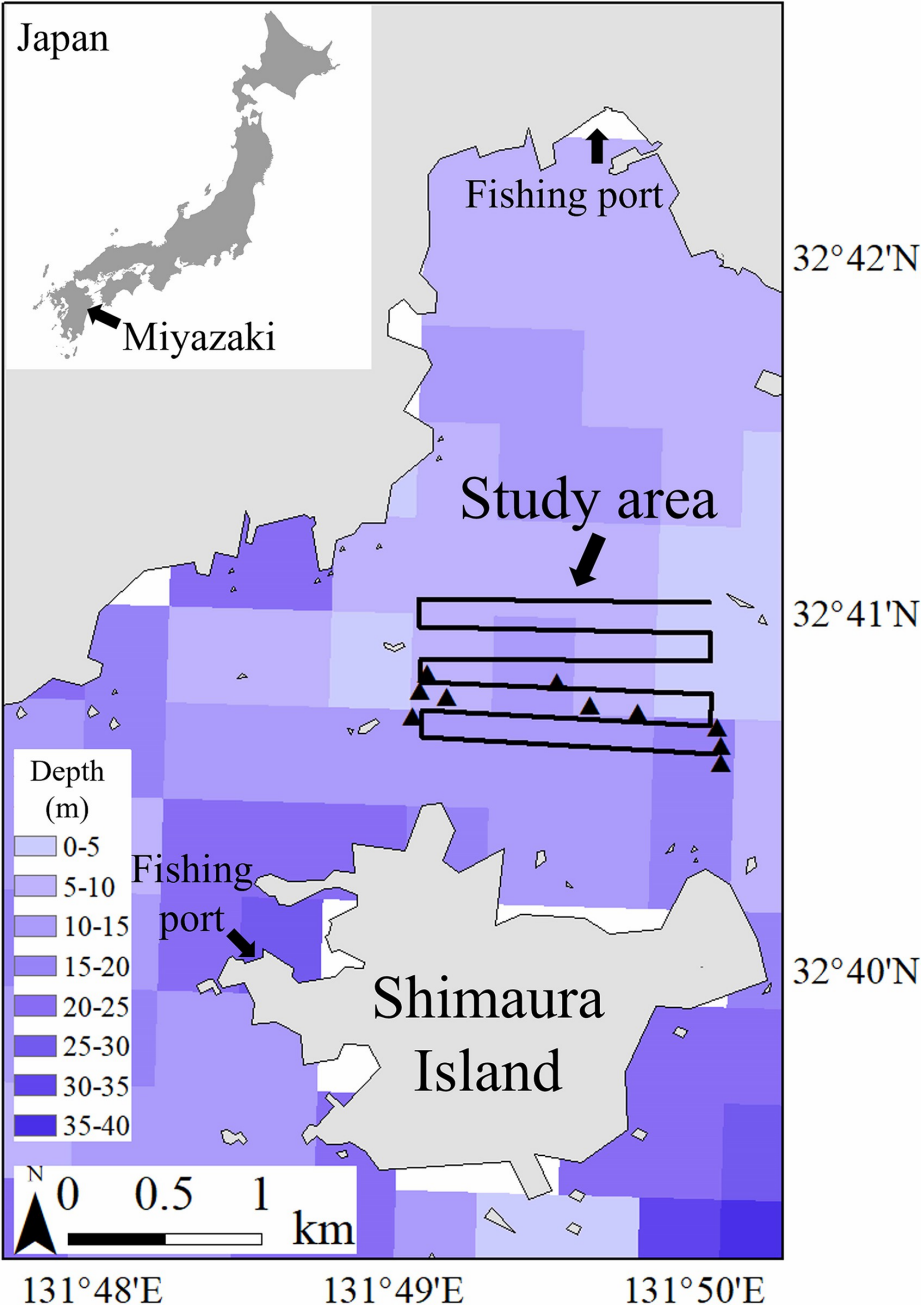
Shimaura Island, Miyazaki, Japan, where the research was conducted. The solid black line represents the survey line, and the triangles indicate points where the sediment survey was conducted.

The survey area was near the fishing port, where fishing boats pass often, and the survey focused on the flat seabed, where the sediment and slope had not changed significantly (Fig 2). The flat seabed was chosen because the seabed backscattering strength is more stable on a relatively smooth seabed than on a rough seabed [26]. The survey lines were set perpendicular or horizontal to the coast, and each measurement line was approximately 1.5 km long. We considered a total of 6 measurement lines in this study and the depth of the survey area was approximately 5 m at the shallowest sites and 30 m at the deepest sites (Fig 2).

In the procedure adopted in this study, we first calibrated the general and quantitative echo sounders, using a calibration ball (a diameter of 38.1 mm tungsten carbide ball), before carrying out acoustic measurements. Since it was necessary to place the calibration ball directly under the transducer, the survey was conducted in an inner bay where waves, wind, and currents were few. The mean wind speed on the day of the survey was 1.6 m/s, and the vessel was anchored from the bow and stern to prevent the vessel from being swept away. During calibration, the calibration ball was hung from 3 points on the vessel’s front, starboard, and port sides with fishing rods so that the calibration ball was directly under the transducer. To confirm that the response from a general echo sounder is linear, the TS of the calibration ball was measured at multiple depths ranging from 9m to 14m. Since the non-split-beam general echo sounder could not measure the position of the target within the beam, the position of the calibration ball was varied within the beam range to find the maximum TS (single observation). The beam width at a low frequency (15 kHz) is 32°, and at a high frequency (200 kHz) is 6°. The maximum TS of the calibration ball was checked using a dedicated tool manufactured by Furuno, and the parameters such as depth, temperature, and salinity (when the maximum TS was detected) were reflected in the recorded data manually. The calibration of the quantitative echo sounder was reflected using an automatic calibration mode [24], and the depth of the calibration ball was roughly 11 m. Then, the Kakutomaru, equipped with the general and quantitative echo sounders traveled along the measurement line and simultaneously recorded the measurements. The vessel speed was maintained at 3~5 knots when making acoustic measurements to prevent the entrainment of bubbles underneath the transducer [27]. Additionally, to compare the backscattering strength of the two echo sounders, the recording conditions of both were standardized, with the pulse width being set as 0.6 ms and the depth range being set as 40 m. Finally, we used a mud sampler (Ekman-Birge seabed sampler 5141-BW, RIGO Japan) along the measurement line [28], to examine the seabed sediments at 10 sites (Fig 2).

### Analysis methods

The first step in the analysis procedure was to verify the accuracy of the general echo sounder that was calibrated using the calibration ball. As a primary verification, the difference in the calibration ball between the measured and theoretical TS was compared at each frequency. As the general echo sounder was not a split beam, we used the maximum TS measured by the calibration ball [7]. In addition, general echo sounders will be used for stock estimation in the future. Therefore, we conducted a secondary validation by comparing the fish abundance measured from the calibrated general and quantitative echo sounders. However, since the frequencies of the general and the quantitative echo sounders used in this study are different, it is difficult to make a comparison using the backscattering strength of the fish school directly. Therefore, we verified the accuracy by comparing the fish numbers calculated using Eq (7) from both echo sounders. After confirming the accuracy of the general echo sounder calibrated with a calibration ball, the data reflecting the calibration values were used to calculate the standard values for the seabed. The standard values were then compared with the data that did not reflect the calibration values to calibrate the echo sounder. Then, Reanalysis was performed using raw data corrected based on seabed backscattering strength. To verify the accuracy of the calibration carried out using the seabed backscattering strength, fish abundances calculated from echosounders calibrated using a calibration ball and seabed were compared.

**Fig 3:**
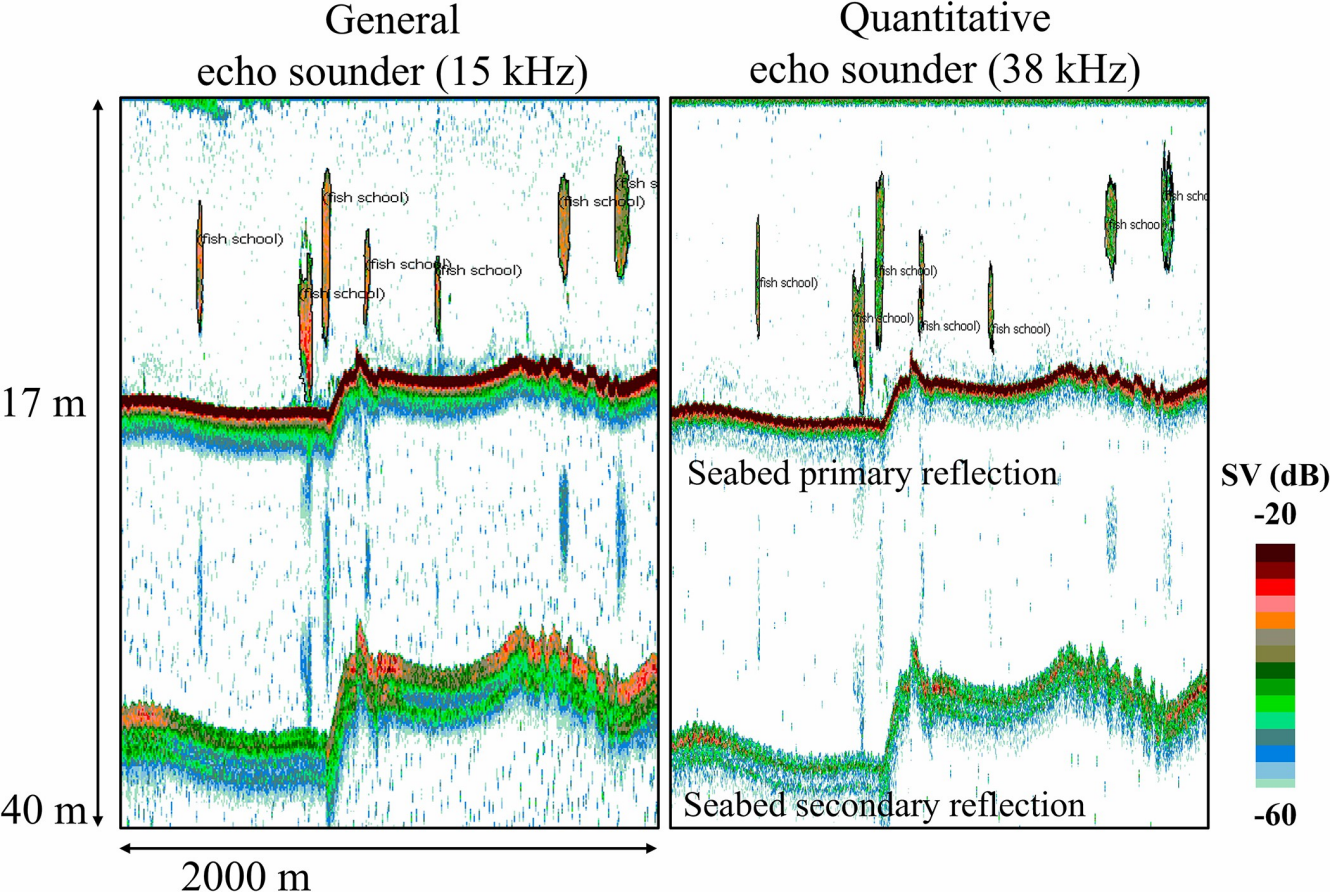
Echograms of the general (15 kHz) and quantitative (38 kHz) echo sounders. The area surrounded by black lines is the fish school of Japanese anchovy detected by Echoview’s school detection function (minimum school length/height: 3 m, maximum vertical/horizontal linking distance in a single fish school: 5 m).

The acoustic data obtained from both echo sounders were analyzed using Echoview ver. 12.1 (Echoview Software Pty Ltd., Australian). S_a_ values were calculated based on the integration of volume backscatter as previously described to determine fish density [16,29]. Since only fish schools were targeted in this analysis, Echoview’s fish school detection function was used to extract the fish schools (Fig 3). The parameters for fish school extraction should be specified based on the length or density of the target species [30]. During our survey period, the target species was the Japanese anchovy (*Engraulis japonicus*), which was around 8.5 cm in body length. Therefore, as parameters for the fish school detection function, the minimum length and height for the candidate fish school were set as 3 m and 5 m was considered as the standard for the maximum vertical/horizontal linking distance in a single fish school. In addition, to eliminate the effects of noise from microorganisms (e.g. plankton and the suspended sediments in the sea), the analysis threshold was set at -60 dB [31], and the areas of weak reflections below this threshold were not included in the analysis.

When the seabed was used in the analysis, determining the seabed line was essential to distinguish between echoes in the water column and seabed echoes. The integration volume immediately below the seabed line was used to calculate the backscattering strength of the seabed. Since there are no scatters stronger than the seabed in the ocean, the depth of maximum S_V_ in each ping can be considered as the seabed [32]. In addition, since the TVG function is not equipped with general echo sounders, scattered sound waves are immediately received when the water depth is shallow, and saturation of the backscattering strength can be assumed. Therefore, secondary reflections on the seabed were also considered in this study [33–35]. The maximum echo from a candidate range of ±1 m from a water depth twice that of the seabed was considered as the seabed secondary reflection [36,37]. However, as the recording range was 40 m if the candidate range exceeded 40 m, we treated the record as an error and assumed that secondary reflections of the seabed were not captured.

In the analysis of the seabed, S_v_ values were used as an acoustic indicator. In this study, the ping rates differed depending on the echo sounder used; however, the distances traveled were the same because they were recorded at the same time. Therefore, the horizontal integration range (hereinafter referred to as ‘grid’) when calculating the S_Vmean_ (average of the s_v_ values then converted back into the log measure) was based on the distance. According to previous studies, the grid over which the average can be considered depends on the survey environment and the echo sounder used [35,37–40]. In this study, the S_Vmean_ of the seabed primary and secondary reflections were extracted in 1, 5, 10, 50, 100, and 500 m grids. Additionally, the vertical integration range for S_Vmean_ was 1 m below the seabed primary and secondary reflections for all analyses. Then, the variability of S_Vmean_ extracted in each grid was compared using the chi-square test, with standard deviation and coefficient of variation as criteria. Data visualization was then performed using the “ggplot” package of the statistical programming language R version 4.2.3 (R Core Team, 2023).

Finally, the seabed sediments collected at each site were classified using a simple grain size analysis method, to determine their water content ratios and grain size compositions. The water content ratio is the weight loss after air-drying the sample and is considered an essential preliminary parameter of grain composition [41]. In this study, the grain size boundary of the gravel was 2 mm, the sand was 1/16 mm and less than 1/16 mm was mud [42]. Sediments obtained from the survey were classified using the statistical software KyPlot [43].

## Results and discussion I: Accuracy of general echo sounder calibrated using a calibration ball

### Measured target strength (TS) of the calibration ball

In this study, the sound speed c was 1520.2 m/s, calculated from a water temperature of 21.4°C and a salinity of 30.4 psu. The maximum TS of the calibration ball was −41.3 dB at 15 kHz, measured at a depth of 11.3 m. Using the National Oceanic and Atmospheric Administration’s (NOAA’s) Standard Sphere Target Strength Calculator, the theoretical TS of the 15 kHz calibration ball calculated from the water temperature and salinity measured during the survey was −41.3 dB [44,45]. Therefore, no difference between the measured and theoretical values. At 200 kHz, the maximum value of the calibration ball was −38.8 dB, measured at a depth of 11.5 m. The theoretical TS of the 200 kHz was considered as −39.1 dB [7], and the difference between the measured and theoretical values was as small as 0.3 dB.

### Area backscattering strength (S_amean_) and estimated fish abundance

In the study area, we extracted the data for 45 fish schools as the Japanese anchovy groups. The result of averaging the Sa of those 45 fish schools (S_amean_) was **−**22.5 dB at 15 kHz and **−**17.6 dB at 200 kHz for the general echo sounder, and **−**27.0 dB at 38 kHz and **−**30.0 dB at 120 kHz for the quantitative echo sounder (Table 2). The TS of an 8.5 cm Japanese anchovy at 15 kHz was **−**48.1 dB, calculated using the regression equation for the general TS and body lengths [46]. The TS was **−**47.2 dB at 38 kHz, **−**49.8 dB at 120 kHz, and **−**50.5 dB at 200 kHz [47]. The number of fish calculated using the S_amean_ and TS for each frequency was 554,617 at 15 kHz and 2,401,549 at 200 kHz. For the quantitative echo sounder, the number of fish was 552,090 at 38 kHz and 498,218 at 120 kHz. The number of fish estimated by the general echo sounder showed a small difference from the quantitative echo sounder at low frequencies and a huge difference at high frequencies.

**Table 2:** Area backscattering strength (S_amean_, dB) for all fish schools obtained from each echo sounder and the number of Japanese anchovies (N) in the study area calculated using S_amean_ and the TSs. TS is shown in previous studies [46,47], and N here is the fish density n calculated in Eq 7 multiplied by the area of the fish school.

	S_amean_ (dB)	TS (dB)	N
15 kHz (Cal. using calibration ball)	-22.5	-48.1	5,54,617
15 kHz (Cal. using seabed)	-22.1	-48.1	5,96,233
200 kHz (Cal. using calibration ball)	-17.6	-50.5	24,01,549
200 kHz (Cal. using seabed)	-17.0	-50.5	27,29,540
38 kHz	-27.0	-47.2	5,52,090
120 kHz	-30.0	-49.8	4,98,218

### Accuracy verification of calibrated general echo sounder

A comparison of the measured and theoretical TS of the calibration ball indicated that the difference between the measured and theoretical values was less than 0.3 dB at both frequencies for the general echo sounder. In general, it is suggested that the difference between measured and theoretical TS values should be within 1 dB [7]. Therefore, it is considered that a general echo sounder, which is calibrated with parameters calculated from the maximum TS measurements of the calibration ball, could be used for acoustic measurements.

In terms of fish abundance for the entire study area estimated using S_amean_, the result of 15 kHz frequency for the general echo sounder was less different from the results of both frequencies for the quantitative echo sounder. In particular, the difference with 38 kHz was less than 1%. In general, in the case of the quantitative echo sounders, the frequency often used for resource assessment is 38 kHz, because low frequencies have a wider beam spread and stronger backscattering strength from the fish school than high frequencies [6]. Since there was no significant difference in the overall number of fish at the low frequency of the two echo sounders, we believe it will be possible to calculate the fish abundance using the low frequency of the calibrated general echo sounder. On the other hand, the results of 200 kHz for the general echo sounder were highly overestimated. This is thought to be a result of the strong electrical transmission power [15]. The general echo sounder used in this study was used in the pelagic purse seine fishery, and the transmission power for 200 kHz was set to 2kw to deliver sound waves to deeper depth zones. Instead, it is conceivable that at shallower depths, strong sound waves would be reflected without enough attenuation. The use of too high a power level for the transmission of sound leads to a significant generation of sound at higher frequencies [48]. Therefore, resource estimation in shallow water using the high frequency of general echo sounders is considered necessary to adjust electrical transmit power to the extent that it does not interfere with fishing operations.

## Results and discussion Ⅱ: Determination of seabed backscattering strength for calibration

### Characteristics of volume backscattering strength (S_V_) of seabed extracted from both echo sounders

**Fig 4:**
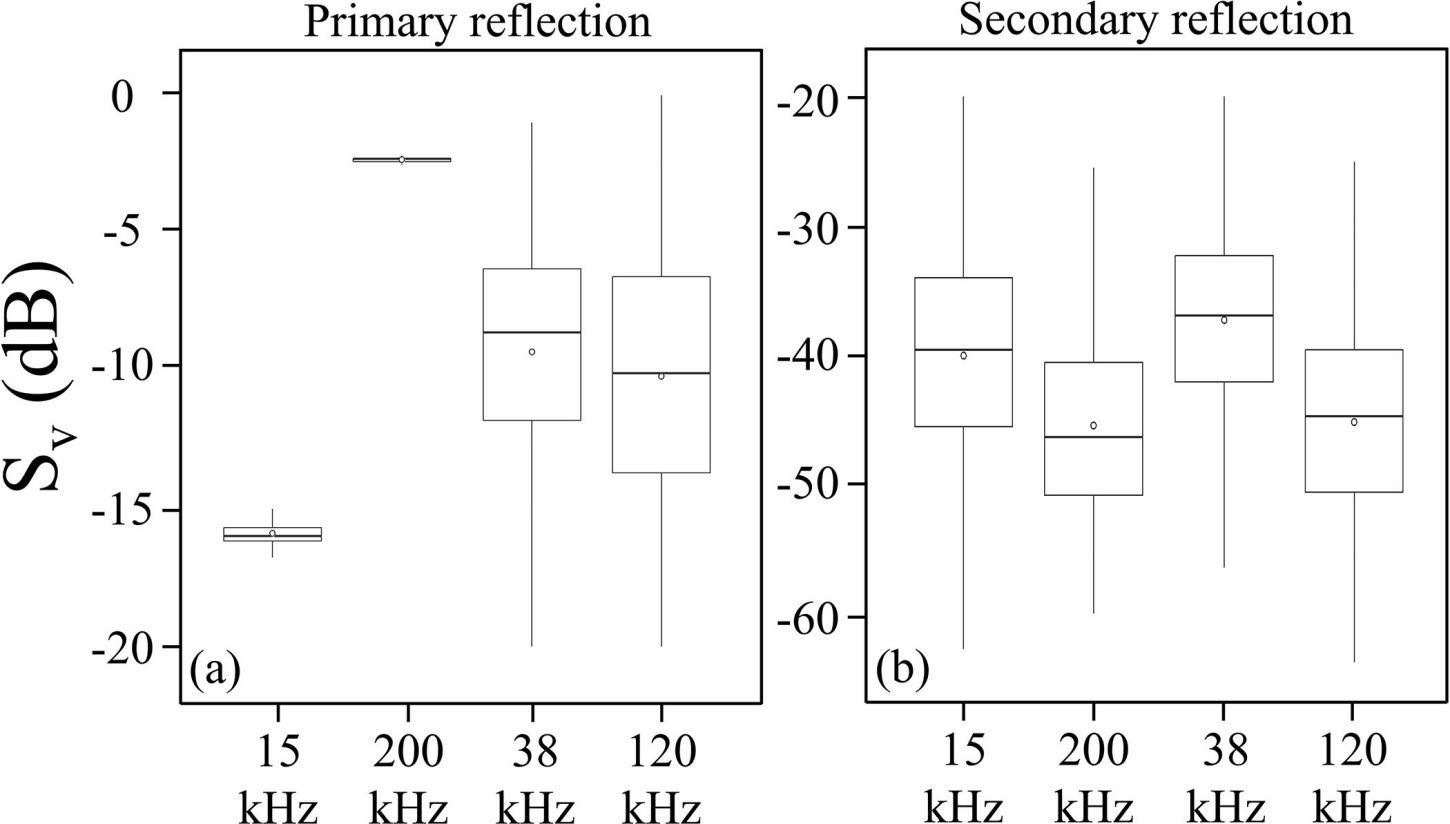
S_V_ of seabed backscattering strength extracted at 1 ping from general and quantitative echo sounders plotted by ggplot. Above and below the solid black lines are the maximum and minimum S_V_ after excluding outliers. The top and bottom edges of the box show the upper quartile and lower quartile, and the middle line shows the median. The white circles indicate mean S_V_.

For the seabed primary reflection, the number of pings extracted from the general echo sounder was 4424. The S_V_ at each ping is concentrated in the range of -16.2 dB to -15.7 dB at 15 kHz and -2.4 dB to -2.3 dB at 200 kHz, portraying no significant changes at both frequencies (Fig 4, Table 3). Unlike the results from the general echo sounder, the S_V_ extracted from the quantitative echo sounder showed a change of about 20.0 dB at both frequencies. Additionally, the number of pings for the seabed secondary reflections extracted from the general echo sounder was 1522, and the S_V_ varied from −82.3 dB to −16.8 dB at 15 kHz and from −59.9 dB to -25.5 dB at 200 kHz. Similar results were observed for the quantitative echo sounder, the S_V_ of seabed secondary reflections varied from −61.5 dB to −12.0 dB at 38 kHz and from -73.2 dB to −23.2 dB at 120 kHz.

**Table 3:** Volume backscattering strength (S_V_, dB) of single echoes extracted from primary and secondary seabed reflections at both echo sounders. SD stands for standard deviation and CV for the coefficient of variation.

	Primary reflection								
		Number of pings	Max.	Upper quartile	Lower quartile	Min.	Mean	SD	CV
General echo sounder	15 kHz	4424	-14.9	-15.7	-16.2	-16.8	-16.0	0.3	-2.0
	200 kHz	4424	-1.9	-2.3	-2.4	-2.5	-2.3	0.1	-3.0
Quantitative echo sounder	38 kHz	42568	-1.0	-2.5	-7.5	-18.0	-5.4	3.8	-69.9
	120 kHz	42568	-1.0	-3.8	-10.8	-23.0	-7.7	4.8	-63.0
	Secondary reflection								
		Number of pings	Max.	Upper quartile	Lower quartile	Min.	Mean	SD	CV
General echo sounder	15 kHz	1522	-16.8	-34.0	-45.6	-82.3	-40.3	9.4	-23.3
	200 kHz	1522	-25.5	-40.5	-50.8	-59.9	-45.4	6.9	-15.2
Quantitative echo sounder	38 kHz	13712	-12.0	-32.2	-42.0	-61.5	-37.2	7.0	-18.7
	120 kHz	13712	-23.2	-39.6	-50.5	-73.2	-45.1	7.3	-16.2

The backscattering strength of the seabed primary reflection obtained from the general echo sounder showed a different trend from those obtained from the quantitative echo sounder. Even though a 20 dB difference in S_V_ was observed for the quantitative echo sounder, more than 75% of the S_V_ for the general echo sounder varied only within a range of 0.5 dB or less. Therefore, it is considered that most of the S_V_ values of seabed primary reflection obtained from the general echo sounders were saturated at both frequencies. In general, sound waves attenuate with the transport distance in the ocean. However, if the water depth is shallow and reflected sound waves from the scatterers are too strong, the reflected sound waves may be recorded before being attenuated significantly [49]. Unlike quantitative echo sounders, the TVG function cannot be automatically executed as a function in the general echo sounder [50]. Because the TVG calculations were manually added to the recorded dataset, it is impossible to adjust the distance attenuation when recording the data, and the backscattering strength of a strong scatterer like the seabed tends to saturate easily at shallow water depths.

On the other hand, it was observed that the S_V_ of seabed secondary reflections obtained from a general echo sounder varied with changes in the seabed. Since the secondary reflections are reflections from the seabed → water surface → seabed, it is more affected by distance attenuation than primary reflections and can more clearly represent changes in the seabed [37,51]. In addition, S_V_ variations of more than 30 dB were observed at all frequencies of a general echo sounder. The reason can be considered that the echo attenuation is associated with vessel pitching and rolling, even assuming that the seabed is uniformly flat. Since slight changes in vessel motion can lead to large variations in seabed echoes, it is considered necessary to use averaged S_V_ values over some range when considering standard values for the seabed scattering strength. From the above, in this study, the standard value used for the seabed scattering strength was taken to be the secondary reflection (rather than primary reflection) averaged over a certain range.

### Seabed sediments and their volume backscattering strength (S_V_) of secondary reflections extracted from general echo sounder

**Fig 5:**
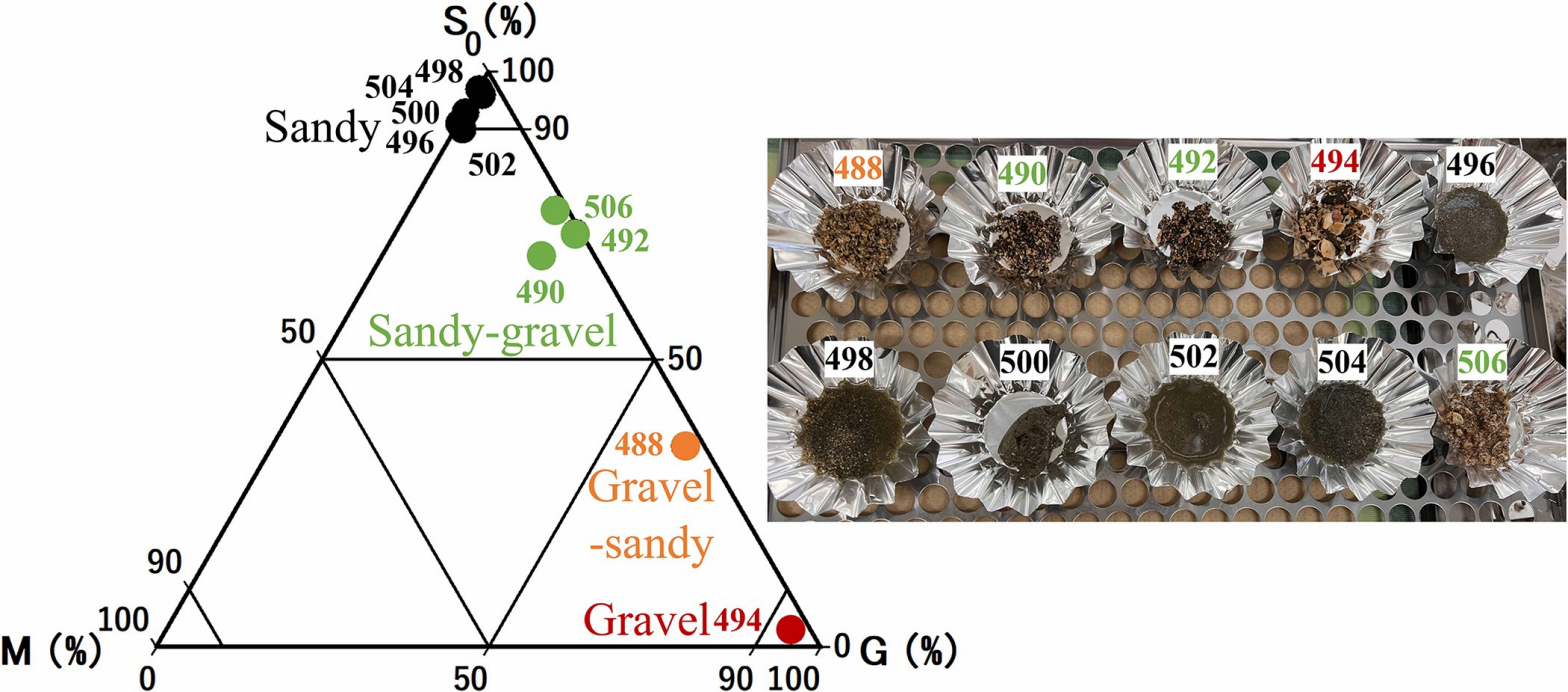
Composition of seabed sediments classified using the KyPlot statistical software. The numbers portray the geographical positioning system (GPS) numbers for the 10 sites sampled. S denotes sand, G denotes gravel, and M denotes mud.

The seabed sediments of the 10 sites considered in this study were classified into 4 major groups (Fig 5). In all survey sites, the sediment properties portrayed a large proportion of sandy sediments (35–97%). In particular, 5 sites (496, 498, 500, 502, 504; sandy) were dominated by sand (>90%). 3 sites (490, 492, 506; sandy-gravel) were dominated by sand, with mixed gravel sediment, with the sand content being 68–76% and gravel content being 22–27%. The remaining 2 sites (488; gravel-sandy, and 494; gravel) were dominated by gravel (>60%).

At these sites, no significant variation characteristics due to grain size composition were observed in the S_V_ values of secondary reflections obtained from the calibrated general echo sounder (Table 4). Certainly, differences in backscattering strength due to differences in seabed sediments are possible [52]. However, in some cases, there was a large overlap between the distributions of the backscattering strength from sand and gravel [8,52]. Especially when the seabed sediment has not changed significantly, as in this study, it was difficult to characterize the seabed backscattering strength. For these reasons, in this study, we used the backscattering strength of the seabed of all the surveyed areas, to determine the standard value to be used for the seabed backscattering strength.

**Table 4:** Grain size composition of the sediment and mean volume backscattering strength (S_V_) of the secondary reflections. S_V_ is the value extracted by 1 ping from the general echo sounder.

GPS number	Gravel (%)	Sandy (%)	Mud (%)	Sediment	15 kHz	200 kHz
488	62	35	3	Gravel‐sandy	‐30.7	‐39.7
490	24	68	8	Sandy‐gravel	‐30.2	‐33.6
492	22	76	2	Sandy‐gravel	‐28.0	‐39.5
494	95	3	3	Gravel	‐41.1	‐40.8
496	0	91	9	Sandy	-30.1	-36.6
498	0	97	3	Sandy	-29.5	-36.3
500	0	93	7	Sandy	-26.8	-41.6
502	1	90	9	Sandy	-39.1	-44.9
504	1	96	3	Sandy	-30.4	-34.1
506	27	72	1	Sandy‐gravel	‐20.4	‐27.4

### Volume backscattering strength (S_Vmean_) of seabed secondary reflections for each grid extracted from general echo sounder

**Fig 6:**
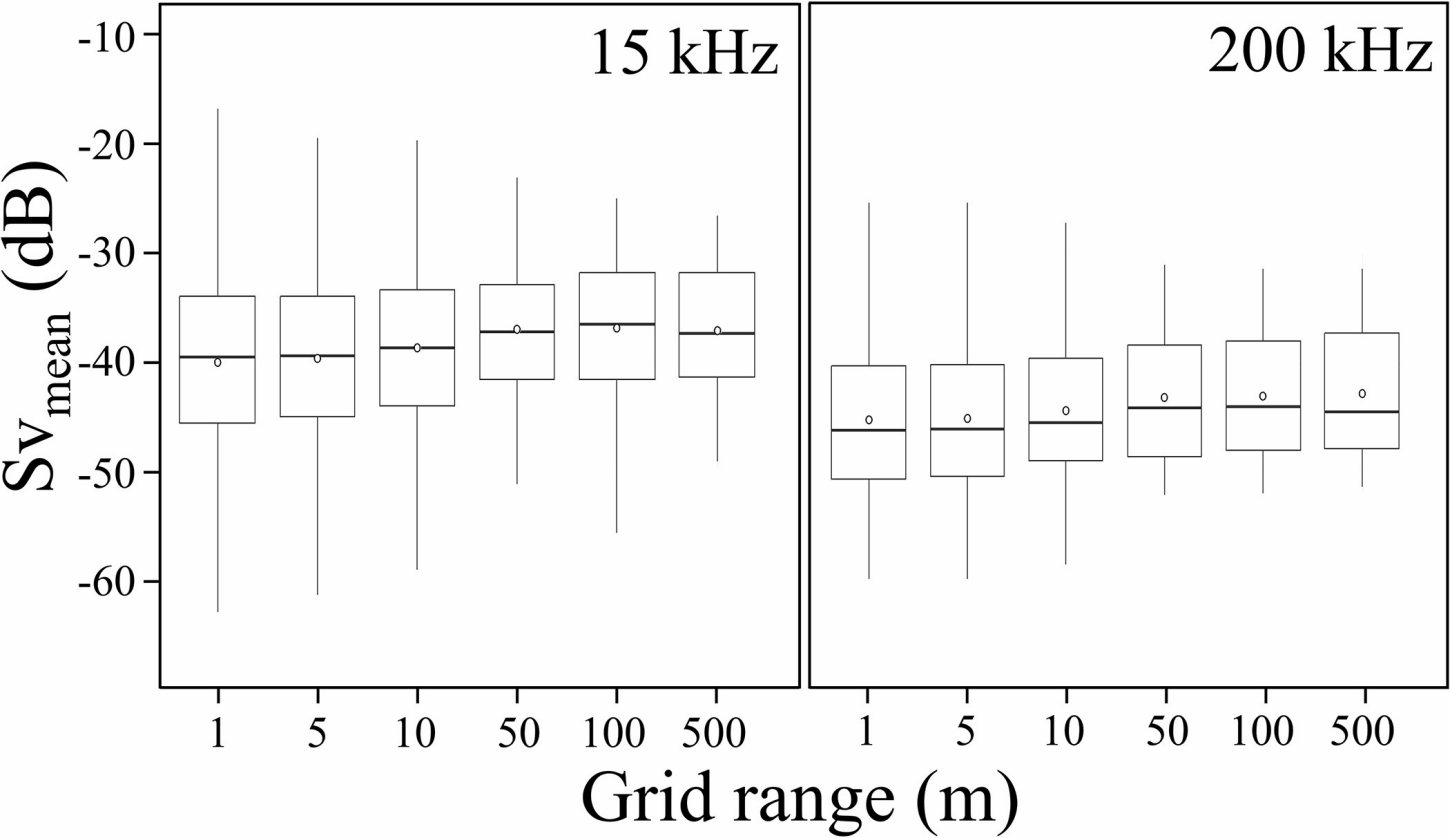
S_Vmean_ of seabed secondary reflections obtained from general echo sounder plotted by ggplot. The S_Vmean_s were extracted in 1, 5, 10, 50, 100, and 500 m grids. Above and below the solid black lines are the maximum and minimum values after excluding outliers. The top and bottom edges of the box show the upper quartile and lower quartile, and the middle line shows the median. The white circles indicate average values.

At 15 kHz, the maximum value of S_Vmean_ did not change significantly with the grid (Fig 6, Table 5). However, the variation between the maximum and minimum values of S_Vmean_ varied from grid to grid. The S_Vmean_ calculated on a 1-m grid showed the greatest variation, approximately 46 dB. The 50-m grid was the boundary, which the S_Vmean_ tended to vary more for narrower grids than wider grids. Additionally, a similar trend was observed at 200 kHz, with the 50-m grid being the boundary. Variation of S_Vmean_ also varied with the grid; however, overall was smaller than 15 kHz.

**Table 5:** Maximum, minimum, and mean values of volume backscattering strength (S_Vmean_, dB), which were calculated from each grid using the secondary seabed reflections obtained from the general echo sounder. SD stands for standard deviation and CV for the coefficient of variation.

	15 kHz					200 kHz				
Grid	Max.	Min.	Mean	SD	CV	Max.	Min.	Mean	SD	CV
1m	-16.8	-63.0	-40.3	9.4	-23.3	-25.5	-59.9	-45.4	6.9	-15.2
5m	-19.4	-61.6	-39.9	9.0	-22.5	-25.5	-59.9	-45.3	6.8	-15.1
10m	-19.7	-58.9	-38.8	8.0	-20.5	-27.3	-58.6	-44.5	6.3	-14.3
50m	-23.1	-51.1	-36.9	6.3	-17.0	-31.1	-52.7	-43.4	5.9	-13.6
100m	-25.0	-55.5	-36.8	6.3	-17.0	-31.5	-52.1	-43.3	5.8	-13.4
500m	-26.6	-55.5	-37.0	6.7	-18.1	-31.5	-51.5	-43.1	5.8	-13.5

**Fig 7:**
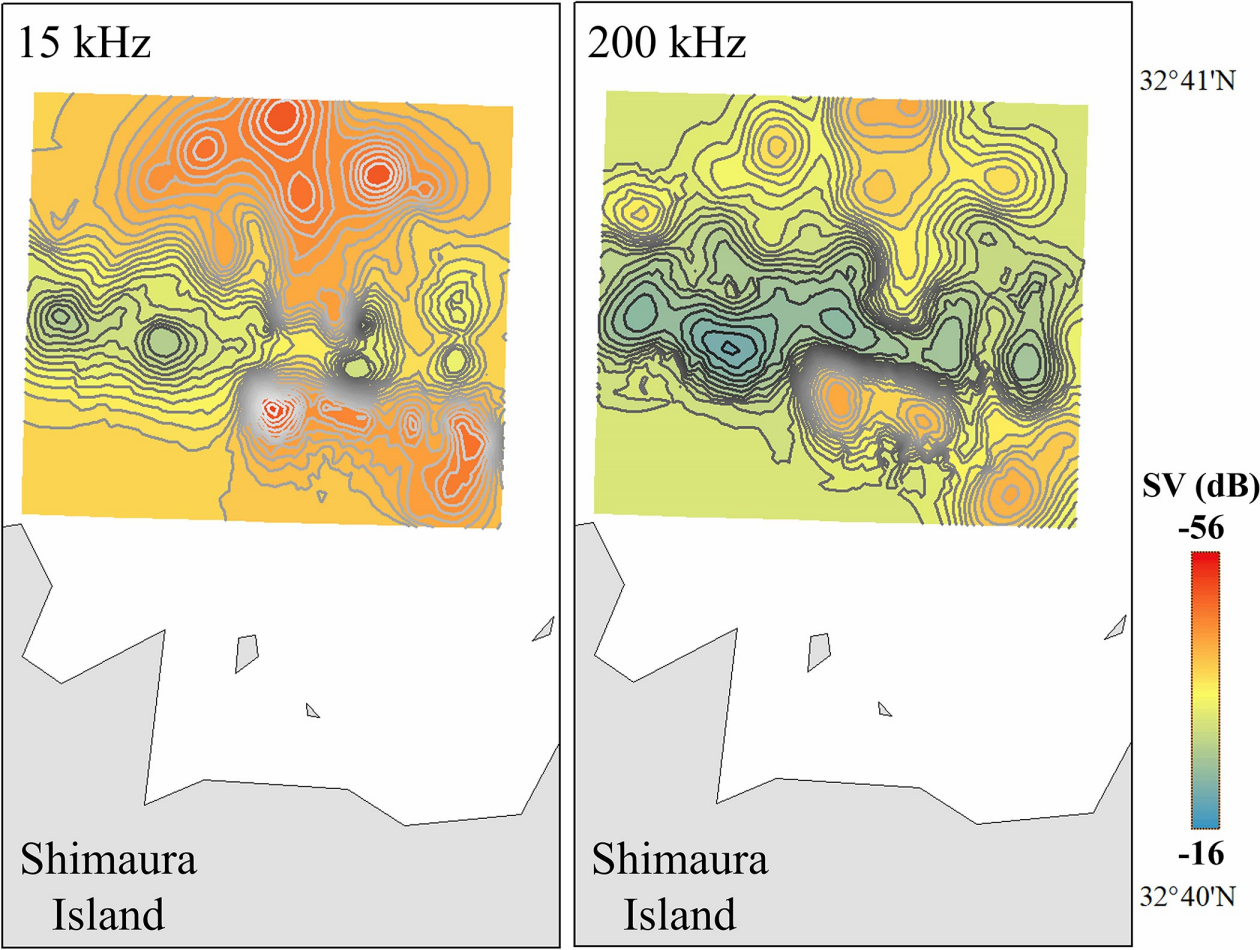
Volume backscattering strength (S_Vmean_) of seabed secondary reflections extracted on a 50m grid. The figures were interpolated using the general kriging function of a spatial statistical method [ArcGIS 10.1, Environmental Systems Research Institute, Inc. (ESRI)].

A possible reason for variation in S_Vmean_ for seabed secondary reflections is attenuation due to surface bubbles generated by the pitching and rolling of the vessel [53]. In particular, the lower frequencies are more susceptible to bubbles, and this may be the reason why the S_Vmean_ at 15 kHz is more varied than the S_Vmean_ at 200 kHz. In addition, the mean value of S_Vmean_ becomes stronger as the grid becomes wider, and the variation is smaller from the 50-m grid. It is suggested that even if there are changes in the seabed sediments, or even if there is vessel pitching and rolling, these effects can be eliminated to some extent if the grid is wider than 50 m. However, setting the grid too wide means that changes in the seabed are largely ignored. To eliminate this tradeoff as much as possible, S_Vmean_ values analyzed on a 50-m grid with a relatively wide grid were used as the standard values of the seabed secondary reflections for calibration (Fig 7).

## Results and discussion III: Demonstration of calibration method using seabed secondary reflections

We calculated the backscattering strength of the seabed secondary reflections from general echo sounder data that did not reflect the calibrated values of the calibration ball. The S_Vmean_ extraction conditions were the same as for the S_Vmean_ used for the standard value. At 15 kHz, the S_Vmean_ had a maximum value of −14.5 dB, minimum value of −47.1 dB, and average value of −28.5 dB; at 200 kHz, the S_Vmean_ had a maximum value of −65.2 dB, minimum value of −86.9 dB, and average value of −77.5 dB. Compared to the standard values of S_Vmean_, there was an average difference of 8.4 dB at 15 kHz and 34.1 dB at 200 kHz. This difference was used to correct and reanalyze the data from the general echo sounder, which did not reflect the calibrated values.

Then, the accuracy of the calibration using the seabed backscattering strength was verified by comparing the extracted S_amean_ of the Japanese anchovy schools from the general echo sounder calibrated with the seabed and with the calibration ball. The S_amean_ extracted from the general echo sounder calibrated by the seabed backscattering strength were −22.1 dB at 15 kHz and −17.0 dB at 200 kHz. At both frequencies, the differences in the S_amean_ extracted from the general echo sounder calibrated by the seabed backscattering strength and those calibrated by the calibration ball were less than 0.6 dB, which was very small (Table 2).

The results obtained from the general echo sounders calibrated using the seabed backscattering strength and the calibration ball did not differ significantly. Therefore, we could verify that the calibration method carried out using the seabed was effective. However, as discussed in the previous section, the high-frequency transmission power used in this study was made stronger to search for fish in deeper waters. Therefore, calibration performed by two methods may not correctly correct for backscattering strength, resulting in a strong reflection of shallow water and an overestimation of the resource. Additionally, since there was no significant difference in the S_amean_ obtained from the low frequencies of the general echo sounder calibrated with the calibration ball and seabed, we believe that the low frequency of the general echo sounder calibrated using the seabed backscattering strength can be used for stock assessment in the future.

## Conclusions

In this study, we analyzed and verified the practicality of a new calibration method carried out using the secondary reflection of the seabed obtained from a general echo sounder. In general, scientific echo sounder calibration using the seabed often uses the backscattering strength of the primary reflection [8–11,23]. However, general echo sounders are not equipped with a TVG function, and the received signal from the primary reflection may be saturated in shallow waters [49,54]. In future applications of general echo sounder calibration using the seabed, the location for setting the seabed standard values should be an area where fishing vessels pass by daily without disturbing the fishermen. In this case, it will be chosen near fishing ports with shallow water depths, and it is difficult to avoid the saturation of primary seabed reflections. Therefore, when using the seabed backscattering strength to calibrate general echo sounders in shallow areas, the use of secondary reflections is preferable to primary reflections. In addition, if the angle between the seabed plane and the horizontal was not smaller than one-half of the beam width, the secondary reflection of the seabed could not be accurately measured [35,55]. In this study, sand and gravel areas were selected where the seabed was not undulating. Nonetheless, the backscattering strength of seabed secondary reflections showed great variability. Therefore, it can be assumed that there would be more variation in rocky areas with large undulations on the seabed. In the future, when calibrating general echo sounders using seabed secondary reflections in other areas, it is important to avoid rocky areas where the seabed changes drastically and consider the areas that are as flat as possible.

There were large variations in the S_V_ values of the seabed secondary reflections at all frequencies. This variation was due to the complex effects of surface bubbles caused by the pitching and rolling of vessel motion and the decrease in echo level due to transducer surface motion. In particular, secondary reflections are more susceptible to these effects than primary reflections. In this study, these effects were reduced by averaging the echo level over a certain range. However, the relationship between the vessel motion and the echo signal is still unclear and is a subject for future study. We considered that the next step is to survey the variation in seabed backscattering strength due to vessel motions subjected to irregular external forces placed in the extremely random phenomenon of ocean waves. In addition, it has been reported that for the same general echo sounders, the measured backscattering strength varies by several dB with changes in seawater temperature [56]. Therefore, another future task in the seabed calibration of general echo sounders is to verify the effect of changes in seawater temperature on the accuracy of the calibration.

In the future, applying the calibration method for general echo sounders using the seabed backscattering strength, the general echo sounders installed on all fishing vessels can be modified to ‘quasi’ quantitative echo sounders. Stock assessment centered on fishing vessels can be established using inexpensive and widely used general echo sounders, instead of using expensive quantitative echo sounders. By using large datasets, we expect that there will be a smooth transition in the fisheries industry, from adopting methods based on intuition and experience to those based on scientific data.

## Supporting information

S1 Table Seabed volume backscattering strength (S_V_) extracted at a single ping was obtained from the calibrated general echo sounder. (XLSX)

S2 Table Area backscattering strength (S_a_) values and the number of Japanese anchovies (n) obtained from each fish school. (XLSX)
