# Supplementary material for: Calibration of commercial fisheries echo sounders using seabed backscatter for the estimation of fishery resources
Source: PLoS One. 2024 May 10;19(5):e0301689. doi: 10.1371/journal.pone.0301689 (PMC11086921; doi:10.1371/journal.pone.0301689)
Supplement: Attachment — Submitted filename: Response to Reviewer 2.docx [file pone.0301689.s003.docx]

Response to Reviewer 2

**We have made major changes throughout the text. Terms were clarified and long sentences were shortened. Also, as you pointed out, there is not enough statistical analysis, and showing only averages is very lacking. Therefore, we have added statistical discussion on the results and discussion using standard deviation and coefficient of variation. Furthermore, we have modified and added diagrams to make the text clearer.**

1. **Abstract** L22. Change semicolon by full stop. L24. I feel “quantification of fisheries echo sounders quantitatively” to be redundant. L27. “Therefore” is not necessary here. L28. I suggest changing “The method involves using” by “Our approach is based on the usage of”. L31. The semicolon is not necessary: “...the seabed echoes, which were calibrated by comparing…”

**We have revised the abstract as commented.**

2. **Introduction** L44. Are being depleted. L49. I suggest to substitute “fishes” by “biomass present”. L51. I suggest to substitute “quantify” by “estimate”.

**We have revised the text as commented.**

3. **Introduction** L52. “Several disadvantages”, but only one is mentioned.

**We have changed the sentence as follows “Although quantitative echo sounders can estimate the backscattering strength of an object, they have several disadvantages, e.g., the echo sounders have limited survey coverage because they are generally installed on large vessels and have limited survey numbers because they require specialized knowledge”.**

4. **Introduction** L63-65: This sentence is not clear. Please, clarify.

**We have changed the sentences as follows “In order to attribute quantifiability to general echo sounders, it is important to compensate for the strength of the reflected sound. Therefore, it is essential to calibrate the transducer system that is processing the sound wave”.**

5. **Introduction** L74: I suggest to substitute “Due to” by “For”. L80. I suggest to substitute “measure the backscattering strength of the seabed” by “the backscattering strength of the seabed can be measured”.

**We have revised the text as commented.**

6. **Introduction** L76-77. I feel “In this method, we used the backscattering strength of the seabed” to be redundant looking at how the previous sentence ended.

**We have simplified the text as follows “Therefore, in this study, we considered a direct calibration method using the seabed, which does not change significantly temporally or physically, instead of a calibration ball”.**

7. **Introduction** L84. What is “inter-ship calibration”? It has not been defined.

**We have added the explanation about the inter-ship calibration as follows “When using multiple general echo sounders in the same area, it is important to compensate between vessels to obtain the same measurement level. By using the same seabed, inter-ship calibration can be easily performed”.**

8. **Methods** L91. Change semicolon by full stop. L106. Change semicolon by full stop. L110. The equation in the text are subjected to the same puntuaction rules, so a full stop or a comma (as in this case) must be placed at the end of equation (1). L115. I suggest to substitute “; therefore, it is” by “, so it is”. L121. It find referencing an equation that does not already appear in the text a bit weird. I suggest to substitute “using Eq. (2) [17]” by “as” and move the reference [17] after describing the variables. L149. Same comment as in L121. L150. The full stop is missing. L152. Change semicolon by full stop. L155. The full stop is missing.

**We have revised the text as commented.**

9. **Methods** L122. The final comma is missing. L123-125. I suggest to describe all the parameters involved in Eq. (2) just after the equation, and then the authors may point out related observations such as “the factor of transmit and received” and the characteristic of R^{-2}. L128. What does “with the characteristic of exp(-2\alpha R)2 mean? L130. Equation (3) is not an equation, as there is no equality. Moreover, the authors must mention in what basis are the logarithms expressed.

**We have changed the sentence as follows “Where** **P_o_ (Pa) is the output transmission sound pressure at the transducer terminals amplitude normalized to the 1 m range, M (V/Pa) denotes the receiver’s sensitivity, G_R_ is the gain of the front-end amplifier, R (m) is the propagation distance, α (dB/km) is the absorption attenuation coefficient, b is the beam pattern (function of direction describing the amplitude sensitivity), and ts is the linear measure of the backscattering cross-section σ_b_. The ‘P_o_MG_R_’ is called the factor of transmit and receive (denoted by K_TR_), which depends on the transducer used and is determined by calibration. In addition, the sound wave spherically diverges with the R^-2^, and with it, when the sound waves absorb energy from the components of seawater, the press of a sound wave decreases exponentially with the distance along the propagation path with exp(-2αR). The TVG calculations of the distance attenuation items included in Eq. (2) yield a logarithmic distance characteristic of 40logR + 2αR. Using this distance characteristic to decibel-converted Eq. (2), we calculated the TS using the following equation”.**

10. **Methods** L133. K_{TR} was used in L125, but KTR is used in this line.

**We have added the sentence in the text as follows “where TS is 10 log ts, EL is 10 log E_ts_, KTR is 10 K_TR_”.**

11. **Methods** L153. Again, this is not an equation. Please, describe precisely the relation between the parameters.

**We have changed the sentence to “Substitute Eq. (5) into Eq. (4) and the distance attenuation terms include the propagation distance R were subjected to TVG processing, yielding a logarithmic distance characteristic of 20logR + 2αR”.**

12. **Methods** L102-103. I suggest to omit the formulas in Fig 1, as they are commented afterwards, so the footnote fully describes the image. L115. What are single echo and group echoes? Please, clarify. L138-140. P_{0} and R have already been described, and the rest of the parameters are described below. I strongly recommend the authors to better explain the images instead of defining the parameters which are detailed in the text.

**We have added detailed explanations to the text in Figure 1 and Figure 2.**

13. **Methods** L156-159. I suggest the authors to motivate better why SA is preferred over SV.

**We have added the sentence in the text as follows “Because most echo integrators provide data in terms of integration over one layer, SA is an important parameter in fisheries acoustics. This quantity is a measure of the energy returned from a layer between two depths and defined as the integral of SV with respect to depth through the layer”.**

14. **Methods** L158. I suggest to substitute “Notably, SA” by “This quantity is”. L161. I suggest to add “in Eq. (4)” after “TS”.

**We have revised the text as commented.**

15. **Methods** L160. The R1 and R2 are not defined.

**We have added the sentence in the text as follows “Where sa is the area backscattering coefficient, SA and SV are the log measure of sa and sv, R1 and R2 indicate the depth to be integrated into the vertical direction”.**

16. **Methods** L161. The number of target species is denoted as “n” in the following section. The authors should consider to include this notation here.

**We have added the “n” in equation (7) and changed the sentence to “Dividing the calculated value by the ts, which is the target strength of one scatterer (a linear measure of σ_bs_) in Eq. (3), the number n of the target species is calculated, and the distribution density per area within a specific sea area is also obtained”.**

17. **Methods** L166. I do not support the usage of Figure 3, since it does not add any value to the manuscript.

**We have put Figure 3 and Figure 6 together. And we have added a box diagram as Figure 5 that allows visualization of the SV_mean_ variation.**

18. **Methods** L167. I suggest removing “in this study” since it is unnecessary here. L185. Change semicolon by full stop. L185. Add “in this study” after “measurement lines2. L185. A linking word is missing before “The depth…”. “Besides that,” suits well here.

**We have revised the text as commented.**

19. **Methods** L199. There are sentences where low numbers are in letter and others that are in numbers. I suggest taking the same criterion everywhere (preferably, with letter. i.e., “ten” instead of “10”). L360. Replace “10” by “ten”. L370. Replace “10” by “ten”.

**We have changed all letters to the numbers.**

20. **Methods** L229. Change semicolon by full stop. L236. Change semicolon by full stop. L241. I suggest rephasing this sentence as “We treated the record as an error…”. L242. I suggest rephasing this sentence as “assumed that secondary reflections of the seabed were not captured…”. L255. The authors of the references were not named until this point (Wentworth). Please, take the same criterion everywhere. L256. I suggest changing “based” by “depending”.

**We have revised the text as commented.**

21. **Results and discussion** L265. State the water temperature and salinity.

**We have added the sentence in the text as follows “In this study, the water temperature was 21.4°C, the salinity was 30.4 psu, and a 38.1 mm WC sphere gives a sound speed c of 1520.2 m /s”.**

22. **Results and discussion** L265. A linking word is missing before “There was…”. “Therefore” suits well here. L284. The authors of the references were not named until this point (Amakasu et al). Please, take the same criterion everywhere.

**We have revised the text as commented.**

23. **Results and discussion** L297. The authors say the differences were low, but there are a 110000 difference between 4th row and 6th row in Table 2, which I think is not negligible.

**We have changed the sentence to “****The fish numbers estimated using both the echo sounders indicated that the difference in the abundances was low at low frequencies, and the difference increased at higher frequencies”.**

24. **Results and discussion** L303-306. This four-line sentence is difficult to read. Consider rephrasing it.

**We have changed the text to “A comparison of the measured and theoretical TS of the calibration ball indicated that the difference between the measured and theoretical values was less than 0.3 dB at both frequencies, for the general echo sounder. The main factors affecting the calibration ball TS are the sound speed, frequency, and radius of the calibration ball. When frequency and calibration ball are fixed, the sound speed calculated from the water temperature and salinity of each study area has the most influence on the calibration ball TS. Since The assumed range of c is from 1470 to 1550 m/s calculated from Japanese coastal conditions, it is possible to calibrate echo sounder systems by these fixed values to an accuracy of 0.5 dB. In this study, since the difference between the measured maximum TS and theoretical TS of the calibration ball was less than 0.5 dB, the general echo sounder calibrated using the parameters calculated from the maximum measured TS of the calibration ball was found to be an echo sounder that could be used for acoustic measurements”.**

25. **Results and discussion** L322. What is the echo number?

**We have changed the echo number to the number of single echoes.**

26. **Results and discussion** L336. Change semicolon by full stop. L403. Change semicolon by full stop. L406. I suggest to substitute “; however,” by “, although”. L411. Replace “1” by “one”. L416. I suggest to move the reference [52] just after “According to a previous study...”.

**We have revised the text as commented.**

27. **Results and discussion** L374-375. I do not understand “The difference in acoustic impedance of the medium is large, the reflection is large”. L387. What does “acoustic impedance”?

**We have changed the sentences as follows “At these sites, no significant variation characteristics due to grain size composition were observed in the SV values of secondary reflections obtained from the calibrated general echo sounder. This may be due to differences in reflection characteristics and echo convergence time for different acoustic impedances. Since sand particles are sufficiently smaller than the underwater wavelength, the reflection of sound waves is close to specular reflection. In contrast, if gravel, particles larger than the underwater wavelength, is mixed in, the diffuse reflection will occur. Therefore, the maximum value of seabed backscattering strength was considered to be greater in sandy sediments, compared to gravelly sediments”.**

28. **Conclusions** L482. Replace “½” by “one-half”. L490. Change semicolon by full stop.

**We have revised the text as commented.**
