# Supplementary material for: Calibration of commercial fisheries echo sounders using seabed backscatter for the estimation of fishery resources
Source: PLoS One. 2024 May 10;19(5):e0301689. doi: 10.1371/journal.pone.0301689 (PMC11086921; doi:10.1371/journal.pone.0301689)
Supplement: Attachment — Submitted filename: Response to Reviewer 3.docx [file pone.0301689.s004.docx]

1. Consider a title that notes the use of seabed backscatter

**We have changed the title to [Calibration of Commercial Fisheries Echo Sounders Using Seabed Backscatter for the Estimation of Fishery Resources].**

2. Line 39, specify what is meant by “them”, i.e., change to “resource”. Line 43, “scientifically” is not needed. Line 48, “short time” is unclear, consider rephrasing that they can efficiently estimate biomass over a wide area. Line 48, Remove “In addition”, not necessary in this statement. Line 55, 2nd and 3rd commas are not necessary. Line 58, move “to survey resources” to the end of the sentence. Line 67, remove “in the sea”

**We made the changes as commented.**

3. Paragraph at line 70, really needs to include a lot of references, and discuss what already is/isn’t being done with regards to seabed calibration. There is lot of work in the seafloor mapping field, plus some work with water column fisheries echosounders (See De Robertis and Hjellvic). They need to develop more on why using the seabed works, which currently has 0 references.

**We have added the information as follows: Calibration methods using seabed backscattering strength have only been validated with scientific echo sounders such as split-beam and multi-beam [References]. Since there is little verification with general echo sounders, which are single beams, it is necessary to examine the validity of the calibration method using the seabed with general echo sounders.**

**Weber TC, Ward LG. Observations of backscatter from sand and gravel seafloors between 170 and 250 kHz. Acoustical Society of America. 2015; 138(4): 2169-218.**

**Eleftherakis D, Berger L, Le BN, Pacault A, Augustin JM, Lurton X. Backscatter calibration of high-frequency multibeam echosounder using a reference single-beam system, on natural seafloor. Marine Geophysical Research. 2018; 39: 55-73.**

**Roche M, Degrendele K, Vrignaud C, Loyer S, Bas TL, Augustin JM, Lurton X. Control of the repeatability of high frequency multibeam echosounder backscatter by using natural reference areas. Marine Geophysical Research. 2018; 3: 89-104.**

**Hjellvik V, Robertis AD. Vessel Comparison on the Seabed Echo: Influence of Vessel Attitude. NOAA Technical Memorandum. 2007; NMFS-AFSC-171.**

4. Line 94, What is meant by “upgrade”?

**We have changed the text to [Therefore, the echoes obtained from a general echo sounder can be calculated and the data can be quantified].**

5. Line 92, Replace distance with range throughout this paragraph. What is the ‘essential role’? Clarify what is meant here. Line 98, TVG does not improve the reception sensitivity, it amplifies the received signal as a function of range. Clarify.

**We have changed the text to [The TVG function is to compensate for the range dependence of the echo. When echoes propagate through seawater, they undergo diffuse attenuation and absorption attenuation. Diffuse attenuation is the attenuation due to spherical diffusion, with the characteristic of R^-2^ for the range R (m). Absorption attenuation is the attenuation due to energy absorption by seawater’s components. It is exponential with the exp(-2αR) characteristic for the absorption attenuation coefficient α (dB/m). Therefore, TVG processing, which amplifies the received signals as a function for range R, can be used to accurately obtain the echo level of the target].**

6. Line 103/Figure 1, r is range. The proper term is SV¸ where the ‘V’ is subscript. See Maclennan et al., 2002 A consistent approach to definitions and symbols in fisheries acoustics”. Fix throughout.

**We made the changes as commented.**

7. Line 119, The use of 20log and 40log for volume and individual scattering, respectively, needs to be clarified in the paper. Are you doing both, and if so, provide the equations.

**We have changed the text as follows: Since the TVG function differs for single and multiple targets, 40logR TVG was used for single target calculations and 20logR TVG for multiple targets calculations in this study.**

8. Line 130, σbs is linear, clarify and replace ‘ts’ in equation 2 with σbs. Fix throughout. Line 139, Again, use σbs in place of ‘ts’, as is consistent with MacLennan et al., 2002.

**We made the changes as commented. We also have changed the text to explain equation 2 as follows: Target strength (TS, dB) is a logarithmic measure of the area backscattered by a single target. It is normally described by the backscattering cross-section σ_bs_, which is a meaningful parameter in linear measure. The scattered sound pressure wave P_ts_ (Pa) from the target returns to the transducer under the influence of two types of attenuation and beam pattern. Then, the transducer converts scattered sound signals into electrical signals and sends them to the preamplifier. In the preamplifier, the receiver sensitivity M (V/Pa) and the gain of the front-end amplifier GR are used to output the voltage E_ts_ (V).**

9. Line 156/Equation 4, Proper use is subscript of the V, as in sV.

**We made the changes as commented.**

10. Figure 2, This figure indicates the basic concepts and terminology used in fisheries acoustics and seems to be excessive to include in this manuscript. In addition, these figures already exist in common acoustic literature. This one in particular looks similar to Figure 1 and Figure 2 in Furusawa 2021. Consider removing or citing previous work instead.

**We removed Figure 2 and cited previous studies instead.**

11. Paragraph at 167 and equation 7, The area backscattering coefficient is sa and the units here are incorrect, again see MacLennan et al., 2002.

**We have changed the text to [Additionally, as s_v_ was proportional to the distribution density per unit volume, the final value used in the resource calculation was considered as the distribution density per unit area backscatter coefficient (s_a_, m^2^/m^2^)].**

12. Line 169, This sentence is unclear. What does layer number have to do with sA? This statement can be removed. Line 174, R1 and R2 represent the lower and upper ranges of the volume over which sV is being integrated. Clarify this sentence.

**We made the changes as commented.**

13. Line 175, ‘ts’, as a lower case form, is not a valid representation of the linear form of target strength. Target strength is a logarithmic unit, thus σbs is the linear form. The parenthetical here is incorrect. Line 176, This is confusing, if you correctly give the units for sa (m2 m-2) then you can clarify that the resulting unit when dividing by σbs (which has units of m2) is target species per unit area.

**Sorry for the confusion. We have newly defined all parameters and units etc. as commented.**

14. Figure 3, What are the axes on the seabed backscattering strength maps on the right? Labels are necessary to interpret these. Label the subfigures as (a), (b), and (c) to be able to directly reference them in the text. Line 189, The figure caption needs to include specific details defining ‘seabed backscattering strength’. Remove the term ‘acoustic indicator’ and refer specifically to SV when discussion volume backscattering strength. The seabed maps look interpolated. Are they? Specify in the caption. Line 199, Figure 3 does not substantiate the claim that the sediment and slope had not changed significantly, and there is no indication of bathymetry. Consider adding bathymetric contours to the Sv representations in Figure 3.

**The seabed backscattering strength maps is separated from this figure because it would be confusing if it were shown in this figure. We have also added bathymetry information and mud sediment sites information to the figure (changed Figure 3 to Figure 2).**

15. Line 205, Remove ‘Besides that’.

**We made the changes as commented.**

16. Line 212, What depth was the sphere suspended? Were multiple depths used to confirm the linearity of the response from the general echosounder? Line 218, Define ‘peak’ here, which should be referring to the peak backscatter or target strength of the calibration sphere. Line 221, What are the parameters?

**We have added the information about the calibration in general echo sounder as follows: To confirm that the response from a general echo sounder is linear, the TS of the calibration ball was measured at multiple depths ranging from 9m to 14m. Since the non-split-beam general echo sounder could not measure the position of the target within the beam, the position of the calibration ball was varied within the beam range to find the maximum TS. The maximum TS of the calibration ball was checked using a dedicated tool manufactured by Furuno, and the parameters such as depth, temperature, and salinity (when the maximum TS was detected) were reflected in the recorded data manually.**

17. Line 217, Replace ‘know’ with ‘measure’. Line 218, Remove ‘with a wide beam’ and ‘with a narrow beam’.

**We made the changes as commented.**

18. Line 225, “to prevent recording the waves reflected from air bubbles”. Does this refer to preventing entrainment of bubbles underneath the transducer? Please clarify.

**We have changed the text to [The vessel speed was maintained at 3~5 knots when making acoustic measurements to prevent the entrainment of bubbles underneath the transducer].**

19. Line 229, Include sediment sites in the map on figure 1.

**We made the changes as commented (included in Figure 2).**

20. Line 235, Did you use a single observation of the maximum TS observed or a mean of an ensemble of observations? Report the number of TS measurements used to calculate the on-axis measurement.

**We used a single observed maximum TS observable.**

21. Line 241, Please elaborate what is meant by this sentence. Line 243, 250, It is unclear how these different calibrations were applied in the processing. Were the echosounder observations corrected afterwards using an offset? Or were data reprocessed using a modified gain based on the observed target strength/seabed measurement? Please expand.

**We have changed the text to [After confirming the accuracy of the general echo sounder calibrated with a calibration ball, the data reflecting the calibration values were used as the standard values for the seabed. The values of seabed backscattering strength were then compared with the data that did not reflect the calibration values to calibrate the echo sounder. Then, Reanalysis was performed using raw data corrected based on seabed backscattering strength. To verify the accuracy of the calibration carried out using the seabed backscattering strength, fish abundances calculated from echosounders calibrated using calibration ball and seabed backscattering strength were compared].**

22. Line 252/Figure 4, Please include at least the depth/range axis and labels on the echograms. Identify the yellow line of the quantitative echogram in the caption. Analysis grid is not defined anywhere in the text. Further, the use of ‘analysis range’ throughout the text is unclear, given the meaning of ‘range’ for volume integration as explained in the methods. Use a different term throughout the text that indicates that this is the horizontal grid.

**We made the changes as commented (changed Figure 4 to Figure 3).**

23. Line 257, The company name for Echoview is currently Echoview Software Pty Ltd. Line 258, ‘ts’ should be σbs. This sentence is redundant with previous methods, simply state that fish abundance was calculated for fish schools based on integration of volume backscatter as previously described.

**We made the changes as commented.**

24. Line 261, Define reaction size and school reactions. Line 266, ‘treated as fish schools’? Simply state were not included in the analysis.

**We have changed the text to [Therefore, the minimum length and height for the candidate fish school were set as 3 m, and 5 m was considered as the standard for the maximum vertical/horizontal linking distance in a single fish school. In addition, to eliminate the effects of noise from microorganisms, the analysis threshold was set at -60 dB, and the areas of weak reflections below this threshold were not included in the analysis].**

25. Line 268, ‘Echoes below the seabed line’ is unclear. Are you referring to the integration volume immediately below the sounder-detected seabed? How was the seabed line detected?

**We have changed the text to [The integration volume immediately below the seabed line was used to calculate the backscattering strength of the seabed, and the S_V_ was used as an acoustic indicator.** **Since there are no scatters stronger than the seabed in the ocean, the depth of maximum SV in each ping can be considered as the seabed].**

26. Line 277-280, These two things do not seem to be related as written. Yes, the pitch/roll of the vessel has a significant impact on the observation of the seabed as a result of changes in the incident angle with the seafloor. Was range then used as an indicator of angle? I.e., were only echoes where the seafloor was a consistent range window averaged, assuming that the observations represented the same vessel position? This is highly sensitive to vessel movement and complete flat seabed structure. Were measurements of pitch and roll available to validate this?

**We removed this part because it was confusing to include the document here.**

27. Line 282, Clarify that this was 1-m below the detected seabed. Line 285, This needs to be defined earlier as it has already been used multiple times throughout the text and figures.

**We made the changes as commented.**

28. Line 286, Earlier it is stated this was done in 50 m grid. Please clarify in either place. Line 290, This information needs to be included in the caption for Figure 1, or Figure 1 should be divided into two figures which can be used in the into/methods (the transect map) and the results (the seabed maps).

**The figures were adjusted because it would be confusing to put the seabed backscattering strength maps before the method section.**

29. Line 286, Also, due to the confusion between terms, is this the power average (average of the linear values, sV, then converted back into log space)?

**We defined S_Vmean_ as follows: the S_Vmean_ (average of the s_v_ values then converted back into the log measure) of the seabed primary and secondary reflections were extracted in 1, 5, 10, 50, 100, and 500 m grids in this study.**

30. Line 294, This is the first time sediments are mentioned. This needs to be described earlier in the survey methods, including how the sediments were collected, and here, additional details into the grain size methods should be included. Define the grain sizes used to distinguish the 3 classes.

**Information on sediment is given at the end of the survey methodology. We used a mud sampler (Ekman-Birge seabed sampler 5141-BW, RIGO Japan) along the measurement line to examine the seabed sediments at 10 sites. This information has also been added to the figure 2. In addition, we also have added the details to the grain size methods as follows: The grain size boundary of the gravel was 2 mm, the sand was 1/16 mm and less than 1/16 mm was mud.**

31. Line 306, Again, include the complete unit for TS here, which is dB re 1 m2

**We defined the unit for TS at the method section as follows: Target strength TS (dB re 1 m^2^, hereinafter referred to as dB).**

32. Line 304-313 As in the methods, clarify if these are single observations of the maximum TS or an average of the highest values, and if so how many observations were included.

**We made it clear at the Method section.**

33. Line 322, use the correct notation, sA, throughout.

**We made the changes as commented.**

34. Line 324-330, sA is not a log unit as being reported. Are these values referring to SV? Line 332/Table 2, Here as well, these cannot be measurements of sA or any areal unit as they are being reported in decibels. Please correct or clarify.

**S_a_ has been reported as Area backscattering strength (MacLennan et al., 2002). We have added information such as units of S_a_ to the method section. In this study, we use S_a_ for all discussions of fish abundance.**

35. Line 338-346, Shouldn’t these estimates be roughly the same at both frequencies for the quantitative echosounder? This indicates that potentially an incorrect target strength values was used. Are these values from the integration using the sphere calibration or the seabed calibration?

**We have revisited the TS. It is indeed strange that the fish abundance calculated from both frequencies of the quantitative echo sounder (calibrated using a calibration sphere) is so different, as you mentioned. We recalculated the abundance one more time using the new TS (Kang et al., 2009). However, both frequencies of the quantitative echo sounder used in this study use different transducers, so there is a slight misalignment in the installation location. Therefore, we think there will be a slight difference in the calculated abundance.**

**(Kang D, Cho S, Lee C, Myoung J, Na J. Ex situ target-strength measurements of Japanese anchovy (Engraulis japonicus) in the coastal Northwest Pacific. ICES Journal of Marine Science. 2009; 66: 1219-1224. doi: 10.1093/icesjms/fsp042)**

36. Line 354, That is a large range with significant implications for the target strength, and it is assumed that the sound speed did not change over the course of the survey. The difference in TS can be driven by many other factors that are internal to the processing of the echosounder. Please discuss here.

**We apologize for the confusion caused by my unnecessary writing. We have corrected the document as follows: In general, it is suggested that the difference between measured and theoretical TS values should be within 1 dB [Reference]. Therefore, it is considered that a general echo sounder, which is calibrated with parameters calculated from the maximum TS measurements of the calibration ball, could be used for acoustic measurements.**

37. Line 360-372, There is currently very little evidence for the claims described in this paragraph outside of the single aggregation of reported values in the paragraph at 339. This needs to be revised or school-by-school results for animal densities need to be included which will remove the variability of comparing different frequencies, from which statistics can be drawn which can include possible error due to both the calibration and target strength values at the differing frequencies.

**We have changed the discussion of this part as follows: In terms of fish abundance for the entire study area estimated using S_amean_, the result of 15 kHz frequency for the general echo sounder were less different from the results of both frequencies for the quantitative echo sounder. In particular, the difference with 38 kHz was less than 1%. In general, in the case of the quantitative echo sounders, the frequency often used for resource assessment is 38 kHz, because low frequencies have a wider beam spread and stronger backscattering strength from the fish school than high frequencies. Since there was no significant difference in the overall number of fish at the low frequency of two echo sounders, we believe it will be possible to calculate the fish abundance using the low frequency of the calibrated general echo sounder. On the other hand, the results of 200 kHz for the general echo sounder were highly overestimated. This is thought to be a result of the strong electrical transmission power. The general echo sounder used in this study was used in the pelagic purse seine fishery, and the transmission power for 200 kHz was set to 2kw to deliver sound waves to deeper depth zones. Instead, it is conceivable that at shallower depths, strong sound waves would be reflected without attenuation. Therefore, we suspect that dense Japanese anchovy schools in shallow areas may have been strongly reflected. Thus, resource estimation in shallow water using the high frequency of general echo sounders is considered necessary to reduce the electrical transmit power or otherwise. In addition, we have summarized the school-by-school results for Japanese anchovy in supporting information file (S2).**

38. Line 377, The difference between -16.8 and -15 dB is still a ~40% difference in backscattering strength. This is considered not significant?

**The results were compared with those of the quantitative echo sounder, and although the quantitative echo sounder showed a change of 5~7 dB, the general echo sounder only showed a change of less than 1 dB. Additionally, the S_V_s are written in the text as the maximum and minimum values; in fact, 90% of the S_V_s were concentrated in the range of 16.2~15.7 dB. We have rewritten this part in the text. We also have changed Table 3 and added Figure 4.**

39. Line 386/Table 3, include the number of observations in the table.

**We made the changes as commented.**

40. Line 392, I would argue that they did (-25 vs -16.8 is a massive difference in the secondary reflection). Clarify.

**We have rewritten this part as follows: On the other hand, it was observed that the SV of seabed secondary reflections obtained from a general echo sounder varied with changes in the seabed. Since the secondary reflections are reflections from the seabed → water surface → seabed, it is more affected by distance attenuation than primary reflections and can more clearly represent changes in the seabed. Particularly, in terms of maximum and mean SV, both echo sounders were found to be larger at low frequencies than at high frequencies. This is because attenuation is more severe at high frequencies than at low frequencies.**

41. Line 399/Figure 5, It has yet to be stated what the intention behind including the data on the secondary reflections is. Why are they included, and what the meaning behind their measurements? Why are you not showing the same figure for the primary bottom, which is a significantly stronger interface and thus the one that should be used for the calibration?

**We have added to the methods section an explanation of why we use secondary reflections of the seabed. In addition, we have added the same figure for the primary seabed (Figure 4).**

42. Line 405/Table 4, Shouldn’t the values for the 50 m grid match shoes reported in Table 3? Why are they not? It is unclear what the difference behind the data being presented are.

**We are very sorry that we do not fully understand the meaning of this question. Table 3 shows the S_V_ extracted by 1 ping each, and Table 4 shows the S_Vmean_ extracted by each grid. Since the units are different, we think the numbers will not be the same.**

43. Line 414, Define saturated here. If the values were saturated, how are the values being presented in the tables variable?

**We have added the information as follows: The backscattering strength of seabed primary reflection obtained from the general echo sounder shows a different trend from those obtained from the quantitative echo sounder. Even though a 20 dB difference in S_V_ was observed for the quantitative echo sounder, more than 75% of the S_V_ for the general echo sounder varied only within a range of 0.5 dB or less. Therefore, it is considered that most of the S_V_ values of seabed primary reflection obtained from the general echo sounders were saturated at both frequencies.**

44. Line 419, What is the implication of the choice of grid size? Expand.

**We have added the information as follows: In addition, S_V_ variations of more than 30 dB were observed at all frequencies of a general echo sounder. The reason can be considered that the echo attenuation is associated with ship pitching and rolling, even assuming that the seabed is uniformly flat. Since slight changes in ship motion can lead to large variations in seabed echoes, it is considered necessary to use averaged S_V_ values over some range when considering standard values for the seabed scattering strength. From the above, in this study, the standard value used for the seabed scattering strength was taken to be the secondary reflection (rather than primary reflection) averaged over a certain range.**

45. Line 424, In the methods, only 3 groups are mentioned. Please clarify either here or in the methods.

**We have fixed the method section.**

46. Line 435, The table does not do a great job demonstrating this. A figure, or including mean Sv on the ternary as a color for each site would be very useful.

**We made the changes as commented.**

47. Line 441, Table 5 does not substantiate this claim. Line 448, The convergence time of the echo is not previously described or any results shown.

**It is indeed as you have pointed out. Therefore, we have decided to stop explaining the details and cite the references. We have fixed the text as follows: At these sites, no significant variation characteristics due to grain size composition were observed in the S_V_ values of secondary reflections obtained from the calibrated general echo sounder. Certainly, differences in backscattering strength due to differences in seabed sediments are possible. However, in some cases, there was a large overlap between the distributions of the backscattering strength from sand and gravel [reference]. Especially when the seabed sediment has not changed significantly, as in this study, it was difficult to characterize the seabed backscattering strength. For these reasons, in this study, we used the backscattering strength of the seabed of all the surveyed areas, to determine the standard value to be used for the seabed backscattering strength.**

**Weber TC, Ward LG. Observations of backscatter from sand and gravel seafloors between 170 and 250 kHz. Acoustical Society of America. 2015; 138(4): 2169-218.**

48. Line 464, Again, what does this refer to? If the values were ‘saturated’, wouldn’t that mean they would be a maximum value? Are you referring to some sort of color or integration threshold? If something is being saturated, how are you collecting measurements?

**Sorry for the lack of explanation. In this study, only the primary reflection of the general echo sounder was saturated. Therefore, the S_V_ value of the secondary reflection of the general echo sounder was used as the standard value. We have changed the flow of the Results and Discussion 2 section to be clearer. First, the primary reflection of the general echo sounder was saturated, so the secondary reflection had to be used. However, the secondary reflections extracted from the 1-ping have a large variation, so we need to use the value averaged over a certain range as the standard value. The backscattering strength of secondary reflections for each seabed sediment then showed no difference in backscattering strength due to seabed sediment. Therefore, we decided to average all the values together instead of per seabed sediment. Finally, we compared the S_Vmean_ values per grid used for calibration.**

49. Line 475-479, This needs to be discussed much earlier in the manuscript to justify the focus of the results on the use of the secondary bottom. It should be explained both in the intro and then reiterated in the methods.

**We made the changes as commented.**

50. Line 484, This, like the previous statement regarding incidence, assumes a lot about the ability of the boat to remain perfectly level and the seafloor to be uniformly flat. Given that it is unlikely that both of things are perfectly true, you likely need to do more to address variability due to the pitch, roll of the vessel. Given the beam angles, minor changes (fractions of degrees) can lead to a lot of variance. There is likely also a tradeoff, as gridding over a larger area means that the likelihood that the seafloor varies is greater as well.

**We discussed this part again as follows: A possible reason for variation in S_Vmean_ for seabed secondary reflections is attenuation due to surface bubbles generated by the pitching and rolling of the ship [reference]. In particular, the lower frequencies are more susceptible to bubbles, and this may be the reason why the S_Vmean_ at 15 kHz is more varied than the S_Vmean_ at 200 kHz. The fact that the variation became smaller from the 50-m grid suggests that even if some parts of the seabed have weak backscattering strength, or even if there is ship pitching and rolling, these effects can be eliminated to some extent if the grid is wider than 50-m. However, setting the grid too wide means that changes in the seabed are largely ignored. To eliminate this tradeoff as much as possible, S_Vmean_ values analyzed on a 50-m grid with a relatively wide grid were used as the standard values of the seabed secondary reflections for calibration.**

**Hamano A, Sanetoh S, Mizutani S, Sumikawa Y, Uchida, K. Effects of ship motion and survey conditions on volume backscattering strength in acoustic survey. Nippon Suisan Gakkaishi. 1988; 54(9): 1533-1543. doi: 10.2331/Suisan.54.1533.**

51. Line 503, So was the same data used to calculate the average as is being presented to indicate the variability in Sv? This is very confusing.

**That is correct. Sorry for the lack of explanation. We have written the [S_Vmean_ values analyzed on a 50-m grid with a relatively wide grid were used as the standard values of the seabed secondary reflections for calibration] at the end of one previous paragraph and fixed this part here as follows: Compared to the standard values of S_Vmean_, there was an average difference of 8.5 dB at 15 kHz and 34.1 dB at 200 kHz. This difference was used to correct and reanalyze the data from the general echo sounder, which did not reflect the calibrated values.**

52. Line 509, From the abstract, I expected this to be the focus of the manuscript, however the results and discussion of this intercalibration and the integration of the anchovy schools is buried to the end of the paper. Reporting Sv across different frequencies is meaningless for a reader to interpret, and instead should focus on the estimated fish density as reported earlier. Rather than using the entire school, layers/intervals within the school can be used to increase the sample number in addition to the dual-frequency estimates of each system. Further, the sphere itself can be integrated to do a similar comparison.

**Due to the structure of this paper, this result and discussion can only be presented here. As you commented, it certainly feels uncomfortable based on the abstract. Therefore, we have made some changes to the abstract. In addition, we have removed the discussion about S_V_ and focused on estimated fish density. However, since the purpose of this study is to examine whether or not map-calibrated general echo sounders will be used in future stock assessments, we believe that the focus should be on stock levels for the entire study area, rather than on detailed comparisons of calibration ball or per fish school. Therefore, we discussed the overall biomass of the entire study area in more detail.**

53. Line 528, This is the first place that the objective of the manuscript is stated and references the secondary seabed. This needs to be stated earlier, and the justification for not using the primary seabed needs to be expanded.

**We made the changes as commented.**

54. Line 540, But the gravel areas and sandy areas show roughly the same variability reported in your results?

**Sorry for the lack of explanation. This study was conducted in a sand and gravel area, not a rocky area. We have rewritten this part as follows: In this study, we analysed and verified the practicality of a new calibration method carried out using the secondary reflection of the seabed obtained from a general echo sounder. In general, scientific echo sounder calibration using the seabed often uses the backscattering strength of the primary reflection. However, general echo sounders are not equipped with a TVG function, and the received signal from the primary reflection may be saturated in shallow waters. In future applications of general echo sounder calibration using the seabed, the location for setting the seabed standard values should be an area where fishing vessels pass by daily without disturbing the fishermen. In this case, it will be chosen near fishing ports with shallow water depths, and it is difficult to avoid the saturation of primary seabed reflections. Therefore, when using the seabed backscattering strength to calibrate general echo sounders in shallow areas, the use of secondary reflections is preferable to primary reflections. In addition, if the slope of the seabed was not smaller than one-half of the beam width, the secondary reflection of the seabed could not be accurately measured. Therefore, in this study, sand and gravel areas were selected where the seabed was not undulating. When calibrating general echo sounders using seabed secondary reflections in other areas in the future, it is important to avoid rocky areas where the seabed changes drastically and consider the areas that are as flat as possible.**

55. Line 544, This is not discussed enough earlier, see previous comments regarding roll. Line 547, Isn’t that the purpose of this study?

**We have made the changes to this　discussion as follows: While there were large variations in the SV values of the seabed secondary reflections at all frequencies. This variation was due to the complex effects of surface bubbles caused by the pitching and rolling of vessel motion and the decrease in echo level due to transducer surface motion. In particular, secondary reflections are more susceptible to these effects than primary reflections. In this study, these effects were reduced by averaging the echo level over a certain range. However, the relationship between the vessel motion and the echo signal is still unclear and is a subject for future study. It is important to survey the variation in seabed backscattering strength due to ship motions subjected to irregular external forces placed in the extremely random phenomenon of ocean waves in the future. In addition, it has been reported that for the same general echo sounders, the measured backscattering strength varies by several dB with changes in seawater temperature [reference]. Therefore, another future task in the seabed calibration of general echo sounders is to verify the effect of changes in seawater temperature on the accuracy of the calibration.**

**Demer DA, Renfree JS. Variations in echosounder– transducer performance with water temperature. ICES Journal of Marine Science. 2008; 65: 1021–1035. doi: 10.1093/icesjms/fsn066.**

56. Line 557, This contradicts the previous paragraph stating that specific requirements need to be met. Line 559, What is meant by error prone? These are not the reasons these have become industry standards. The second clause of this sentence needs a reference, 15 kHz is not as common as 18 kHz.

**We have removed this part of the discussion because it is confusing.**

57. Line 567, This is too general of a statement. Line 568, ‘eco sounders’ should be echosounders. What is meant by ‘automatically’? Expand. Line 567, What is a survey system? Survey of what? Be specific. Clarify or remove as this is better explained in the following sentences.

**Regarding this part, we have removed unnecessary parts and shortened it.**
