# Supplementary material for: Calibration of commercial fisheries echo sounders using seabed backscatter for the estimation of fishery resources
Source: PLoS One. 2024 May 10;19(5):e0301689. doi: 10.1371/journal.pone.0301689 (PMC11086921; doi:10.1371/journal.pone.0301689)
Supplement: Attachment — Submitted filename: Response to Reviewer 1.docx [file pone.0301689.s005.docx]

1. Page 2 (abstract), Line 23 and Page 4, Line 55: Please clarify “at a high frequency” – that the acoustic frequency is high or that data are gathered often? Page 2 (abstract), Line 29: Please clarify “averaged over a certain range” – does this mean over a certain area?

**We have made the changes as commented.**

2. Page 5 Line 81: Please check the sentence: Is it the measurements using a calibrated general echosounder that will be used as a standard value for the seabed in that area?

**That is correct. We have therefore amended the document to read: The standard value for calibration using the seabed is the backscattering strength in a given area measured with a calibrated general echo sounder.**

3. Page 5 Line 83: I advise to specify clearer what “the validation opportunity” could be, e.g., when the fishing vessel has opportunity to pass over an area where a reference value has been established.

**We have made the changes as commented.**

4. Page 6 Line 93: Consider rephrasing the sentence for conciseness. What is it to “calculate an echo”, and what is it to “quantify the data”?

**We have changed the text as follows: Therefore, the backscattering strength of the object targets can be calculated from the echoes obtained by a general echo sounder.**

5. Page 6 line 96, Page 8 line 101: The term “spherical spreading” is preferable over “diffuse attenuation”. Page 6 line 99: If the absorption law is exp(-2αR), the unit of α should be Np/m not dB/m. Page 6 Line 99: Consider removing “Therefore, “. Alternatively, remove the whole sentence since the purpose of TVG was already stated in Line 94.

**We have made the changes as commented.**

6. Page 6 Line 101, Page 15 Line 259, Page 24 Line 383: It is stated in the first location that some recent general fisheries echosounders include TVG. What is the difference between such TVG and what is here referred to as “automatic TVG”? Since this is emphasized as a characteristic difference between quantitative and general echosounders I advise to describe the difference in more length. On Line 259 it is stated that general echosounders do not include TVG, while a different formulation again is used on line 383. Please check agreement throughout the text. Also consider to reduce the number of times this is mentioned.

**We have added the following documents: Unlike the general echo sounder, the TVG function is included in the main unit of the quantitative echo sounder. Therefore, the echoes obtained from the quantitative echo sounder are the values after the TVG calculation. And we also checked the agreement throughout the text.**

7. Page 7 line 122, Page 8 line 130: Please check the sentence, it seems to imply that an area is being backscattered.

**We have changed the sentences to Target strength TS (dB re 1 m2, hereinafter referred to as dB) is a logarithmic measure of the ts, which is the proportion of the incident energy that is backscattered by a single target. The ts is normally described using the backscattering cross-section σbs (m^2^) and distance R (m) from the target.**

8. Page 8 line 136: It may be useful to define two absorption coefficients, or alternatively use 10^(-\alpha R/20) as the amplitude absorption factor throughout. The units of \alpha in this line is dB/m, while the \alpha in Equation (2) is in Np/m if exp means the natural exponential function.

**We have used 10^(-αR/10)^ as the amplitude absorption factor throughout.**

9. Page 8 line 139: Please check if a “log” is missing from “KTR is 10 K_TR.” Page 8 line 139: TS is 10 log (\sigma_bs / r_0^2). Similarly for definitions of logarithmic quantities throughout, please check if reference quantities are missing. Page 9 line 151: Please check the sentence. Can it be divided? Page 14 line 246: Please review the sentence “When comparing …”, which is difficult to follow.

**We have made the changes as commented.**

10. Page 10-11 table 1: It would be helpful for the reader with a brief explanation already here about of these high transmit powers were chosen. Korneliussen et al., ICES Journal of Marine Science, 65(6), 2008, contains recommendations that could be relevant for comparison.

Is the “Beam angle” in the table equal to what is referred to as the beam width in the text? (Often, the beam width is understood as two times the -3 dB beam angle.)

**We have changed the beam angle to the -3 dB beam width in Table 1. In addition, we have added the information about transmit power as follows: Note that the general echo sounder, which is a single beam, was set to a strong transmit power even at high frequency to capture fish school responses in deeper waters. In the case of the general echo sounder, which is a split beam, the transmit power at a higher frequency was lower than at a lower frequency to avoid nonlinear effects.**

11. Page 13 line 223: It is stated that direct comparison between measured target strengths between the general and scientific echosounders is difficult because the measurement frequencies are different. A verification is therefore made by comparing the measurement results in terms of fish abundance. Is this the whole motivation for measuring on fish in the present work? The text should explain clearly how this approach overcomes the problem that the frequencies are different, and why the measurements on fish are desirable in addition to measurements on the calibration ball.

**We have changed the text as follows: In addition, general echo sounders will be used for stock estimation in the future. Therefore, we conducted a secondary validation by comparing the fish abundance measured from the calibrated general and quantitative echo sounders. However, since the frequencies of the general and the quantitative echo sounders used in this study are different, it is difficult to make a comparison using the backscattering strength of the fish school directly. Therefore, we verified the accuracy by comparing the fish numbers calculated using Eq. (7) from both echo sounders.**

12. Page 18 line 290: Please adjust to clarify the calibration state of the echosounder for this first measurement (and for the other results reported). Was it made before any calibration attempts, with factory adjustment values, or after some other form of calibration? Was the initial KTR of the 15 kHz echosounder different from the new value reported at Line 302? Page 24 line 392: Consider to revise the statement “… echo sounders were found to be larger … ”.

**This section is a little confusing and has been removed.**

13. Page 21 line 346: Something seems to be missing from this sentence.

**We have changed the sentence to: The use of too high a power level for the transmission of sound leads to a significant generation of sound at higher frequencies [Reference]. Therefore, resource estimation in shallow water using the high frequency of general echo sounders is considered necessary to adjust electrical transmit power to the extent that it does not interfere with fishing operations.**

**Korneliussen RJ, Diner N, Ona E, Berger L, Fernandes PG. Proposals for the collection of multifrequency acoustic data. ICES Journal of Marine Science. 2008; 65: 982–994. doi: 10.1093/icesjms/fsn052.**

14. Page 22 line 349: Is this section heading meant to encompass all the text until the next numbered “Results and discussion” heading? It appears that not all of it focuses on “1 ping” measurements. For example, consider adjusting the heading text.

**We have made the changes as commented.**

15. Page 27 line 432: This section should discuss why the mean value of S_Vmean increases so strongly with the grid size. Page 27 line 433: It would be helpful with a discussion of why the maximum values are the same for all the grid sizes.

**Since the values in the table include outliers, table 5 has been reworked. We also have included in the document a discussion of why the Sv values increase strongly with the grid size.**

16. Page 31 line 490: This still seems unclear. I have not been able to read from the text exactly what signals saturate the receiver. With “saturation of the fish backscattering strength”, is it meant that echoes from fish saturate the receiver? If so, the text would benefit from a discussion of how this leads to an overestimate of abundance.

**We have changed the text to However, as discussed in the previous section, the high-frequency transmission power used in this study was made stronger to search for fish in deeper waters. Therefore, calibration performed by two methods may not correctly correct for backscattering strength, resulting in a strong reflection of shallow water and an overestimation of the resource.**

17. Page 31 line 492: If the two calibration methods lead to the same abundance measurement results, were the calibration factors almost equal and this result already expected?

**Yes, it is. There was no significant difference in the calibration factors. However, since this study will be used for resource estimation in the future, we think it would be easier to show the difference between the two in terms of fish abundance.**

18. Page 32 line 507: Please revise to clarify what is meant by that the slope of the seabed was not smaller than one-half of the beam width. For example, is the slope the angle between the seabed plane and the horizontal? Perhaps a brief definition of slope could be included where the word is first used. Page 33 line 515: Consider removing the word “While”.

**We have made the changes as commented.**

Page 33: With regard to possible reasons for the observed variations, did you also consider the variability in the first two reflections (bottom and surface) affect the sound beam and thus e.g. the footprint (backscattering area) contributing to the second bottom reflection? How were the weather conditions, with respect to scattering at the surface? Alternatively, would it be possible to reduce the transmit power while calibrating, to be able to use the first bottom echo, combined with a study of the uncertainty resulting from changing the transmit power between high and low? This would of course increase the personnel workload somewhat.

**We also considered the impact of the bottom and the surface. It is quite difficult to discuss this part in this study. Therefore, as a future step, we plan to conduct the same survey in various weather conditions to see how much they affect the calibration. In addition, it is impossible to adjust the transmit power easily. Therefore, another future task is to find a transmit power that is balanced between operation and survey. In the future, we plan to increase the accuracy by conducting more studies.**
