# Supplementary material for: Calibration of commercial fisheries echo sounders using seabed backscatter for the estimation of fishery resources
Source: PLoS One. 2024 May 10;19(5):e0301689. doi: 10.1371/journal.pone.0301689 (PMC11086921; doi:10.1371/journal.pone.0301689)
Supplement: Peer review history

# Peer review: Decision Letter 0

**DOI:** 10.1371/journal.pone.0301689.r001

**Authors:** Vitor Hugo Rodrigues Paiva

30 Jul 2023

PONE-D-23-17337Calibration of Commercial Fisheries Echo Sounders for the Estimation and Management of Fishery ResourcesPLOS ONE

Dear Dr. Zhu,

Thank you for submitting your manuscript to PLOS ONE. After careful consideration, we feel that it has merit but does not fully meet PLOS ONE’s publication criteria as it currently stands. Therefore, we invite you to submit a revised version of the manuscript that addresses the points raised during the review process.

Please submit your revised manuscript by Sep 13 2023 11:59PM. If you will need more time than this to complete your revisions, please reply to this message or contact the journal office at plosone@plos.org. When you're ready to submit your revision, log on to https://www.editorialmanager.com/pone/ and select the 'Submissions Needing Revision' folder to locate your manuscript file.

Please include the following items when submitting your revised manuscript:

- A rebuttal letter that responds to each point raised by the academic editor and reviewer(s). You should upload this letter as a separate file labeled 'Response to Reviewers'.
- A marked-up copy of your manuscript that highlights changes made to the original version. You should upload this as a separate file labeled 'Revised Manuscript with Track Changes'.
- An unmarked version of your revised paper without tracked changes. You should upload this as a separate file labeled 'Manuscript'.

If you would like to make changes to your financial disclosure, please include your updated statement in your cover letter. Guidelines for resubmitting your figure files are available below the reviewer comments at the end of this letter.

If applicable, we recommend that you deposit your laboratory protocols in protocols.io to enhance the reproducibility of your results. Protocols.io assigns your protocol its own identifier (DOI) so that it can be cited independently in the future. For instructions see: https://journals.plos.org/plosone/s/submission-guidelines#loc-laboratory-protocols. Additionally, PLOS ONE offers an option for publishing peer-reviewed Lab Protocol articles, which describe protocols hosted on protocols.io. Read more information on sharing protocols at https://plos.org/protocols?utm_medium=editorial-email&utm_source=authorletters&utm_campaign=protocols.

We look forward to receiving your revised manuscript.

Kind regards,

Vitor Hugo Rodrigues Paiva, Ph.D.

Academic Editor

PLOS ONE

Journal requirements:

When submitting your revision, we need you to address these additional requirements.

1. Please ensure that your manuscript meets PLOS ONE's style requirements, including those for file naming. The PLOS ONE style templates can be found at

https://journals.plos.org/plosone/s/file?id=wjVg/PLOSOne_formatting_sample_main_body.pdf and

https://journals.plos.org/plosone/s/file?id=ba62/PLOSOne_formatting_sample_title_authors_affiliations.pdf

2. We note that you have indicated that data from this study are available upon request. PLOS only allows data to be available upon request if there are legal or ethical restrictions on sharing data publicly. For more information on unacceptable data access restrictions, please see http://journals.plos.org/plosone/s/data-availability#loc-unacceptable-data-access-restrictions.

In your revised cover letter, please address the following prompts:

a) If there are ethical or legal restrictions on sharing a de-identified data set, please explain them in detail (e.g., data contain potentially sensitive information, data are owned by a third-party organization, etc.) and who has imposed them (e.g., an ethics committee). Please also provide contact information for a data access committee, ethics committee, or other institutional body to which data requests may be sent.

b) If there are no restrictions, please upload the minimal anonymized data set necessary to replicate your study findings as either Supporting Information files or to a stable, public repository and provide us with the relevant URLs, DOIs, or accession numbers. For a list of acceptable repositories, please see http://journals.plos.org/plosone/s/data-availability#loc-recommended-repositories.

We will update your Data Availability statement on your behalf to reflect the information you provide.

3. Please include your full ethics statement in the ‘Methods’ section of your manuscript file. In your statement, please include the full name of the IRB or ethics committee who approved or waived your study, as well as whether or not you obtained informed written or verbal consent. If consent was waived for your study, please include this information in your statement as well.

4. We note that Figures 3 and 6 in your submission contain [map/satellite] images which may be copyrighted. All PLOS content is published under the Creative Commons Attribution License (CC BY 4.0), which means that the manuscript, images, and Supporting Information files will be freely available online, and any third party is permitted to access, download, copy, distribute, and use these materials in any way, even commercially, with proper attribution. For these reasons, we cannot publish previously copyrighted maps or satellite images created using proprietary data, such as Google software (Google Maps, Street View, and Earth). For more information, see our copyright guidelines: http://journals.plos.org/plosone/s/licenses-and-copyright.

We require you to either (1) present written permission from the copyright holder to publish these figures specifically under the CC BY 4.0 license, or (2) remove the figures from your submission:

1. You may seek permission from the original copyright holder of Figures 3 and 6 to publish the content specifically under the CC BY 4.0 license. 

We recommend that you contact the original copyright holder with the Content Permission Form (http://journals.plos.org/plosone/s/file?id=7c09/content-permission-form.pdf) and the following text:

“I request permission for the open-access journal PLOS ONE to publish XXX under the Creative Commons Attribution License (CCAL) CC BY 4.0 (http://creativecommons.org/licenses/by/4.0/). Please be aware that this license allows unrestricted use and distribution, even commercially, by third parties. Please reply and provide explicit written permission to publish XXX under a CC BY license and complete the attached form.”

Please upload the completed Content Permission Form or other proof of granted permissions as an "Other" file with your submission.

In the figure caption of the copyrighted figure, please include the following text: “Reprinted from [ref] under a CC BY license, with permission from [name of publisher], original copyright [original copyright year].”

2. If you are unable to obtain permission from the original copyright holder to publish these figures under the CC BY 4.0 license or if the copyright holder’s requirements are incompatible with the CC BY 4.0 license, please either i) remove the figure or ii) supply a replacement figure that complies with the CC BY 4.0 license. Please check copyright information on all replacement figures and update the figure caption with source information. If applicable, please specify in the figure caption text when a figure is similar but not identical to the original image and is therefore for illustrative purposes only.

The following resources for replacing copyrighted map figures may be helpful:

USGS National Map Viewer (public domain): http://viewer.nationalmap.gov/viewer/

The Gateway to Astronaut Photography of Earth (public domain): http://eol.jsc.nasa.gov/sseop/clickmap/

Maps at the CIA (public domain): https://www.cia.gov/library/publications/the-world-factbook/index.html and https://www.cia.gov/library/publications/cia-maps-publications/index.html

NASA Earth Observatory (public domain): http://earthobservatory.nasa.gov/

Landsat: http://landsat.visibleearth.nasa.gov/

USGS EROS (Earth Resources Observatory and Science (EROS) Center) (public domain): http://eros.usgs.gov/#

Natural Earth (public domain): http://www.naturalearthdata.com/

[Note: HTML markup is below. Please do not edit.]

Reviewers' comments:

Reviewer's Responses to Questions

**Comments to the Author**

1. Is the manuscript technically sound, and do the data support the conclusions?

The manuscript must describe a technically sound piece of scientific research with data that supports the conclusions. Experiments must have been conducted rigorously, with appropriate controls, replication, and sample sizes. The conclusions must be drawn appropriately based on the data presented. 

Reviewer #1: Partly

Reviewer #2: Yes

**********

2. Has the statistical analysis been performed appropriately and rigorously? 

Reviewer #1: I Don't Know

Reviewer #2: No

**********

3. Have the authors made all data underlying the findings in their manuscript fully available?

The PLOS Data policy requires authors to make all data underlying the findings described in their manuscript fully available without restriction, with rare exception (please refer to the Data Availability Statement in the manuscript PDF file). The data should be provided as part of the manuscript or its supporting information, or deposited to a public repository. For example, in addition to summary statistics, the data points behind means, medians and variance measures should be available. If there are restrictions on publicly sharing data—e.g. participant privacy or use of data from a third party—those must be specified.

Reviewer #1: Yes

Reviewer #2: Yes

**********

4. Is the manuscript presented in an intelligible fashion and written in standard English?

PLOS ONE does not copyedit accepted manuscripts, so the language in submitted articles must be clear, correct, and unambiguous. Any typographical or grammatical errors should be corrected at revision, so please note any specific errors here.

Reviewer #1: Yes

Reviewer #2: Yes

**********

5. Review Comments to the Author

Please use the space provided to explain your answers to the questions above. You may also include additional comments for the author, including concerns about dual publication, research ethics, or publication ethics. (Please upload your review as an attachment if it exceeds 20,000 characters)

Reviewer #1: This manuscript proposes a simplified echosounder calibration method for commercial fisheries vessels to provide quantitative data for fish stock assessment. The method is based on overpass over seabed areas with known backscattering properties, in place of time-consuming calibration procedures using reference targets, commonly used in fisheries research. The manuscript reports from a large and comprehensive work with interesting and impressive results.

In my opinion, the manuscript is promising but has some important shortcomings in its present form. My recommendation is therefore that a major revision should be carried out before the manuscript may be accepted.

To Review Question 1, the manuscript seems to report from a work that is technically sound, but the manuscript itself needs revision to reach appropriate rigor in its treatment of theory (well known from literature), experiment, and conclusions.

To Review Question 2, there is little statistical analysis in the work. Some details are lacking or need clarification.

To Review Question 3, I found this somewhat difficult to answer. The data are available from the authors “upon reasonable request”, without specification of why they are not openly available online.

To Review Question 4, the manuscript is mostly intelligible and written in standard English, but it would benefit from some improvements.

The authors state that “the calibration of general echo sounders has never been carried out using the backscattering strength of the seabed” (page 6 line 85). In my opinion, the manuscript should account for, and compare with, other uses of the seabed as sonar calibration reference, such as, e.g., Eleftherakis et al., Mar. Geophys. Res. 39 (2018), Weber & Ward, J. Acoust. Soc. Am. 138 (2015), Roche et al., Mar. Geophys. Res. 39 (2018). The reviewer found the two latter references via the Master’s theses of Guimarães (2020), and Cândido (2022).

The manuscript should define the quantities used more precisely and keep clear separation between their natural and logarithmic forms. Several equations need to be corrected and/or explained more clearly in the surrounding text. Details are noted under “Detail comments” below. A thorough foundation for the equations built upon in the manuscript are given in the report series “Fisken og havet” (The Fish and the Sea), available online at Institute of Marine Research (https://www.hi.no/en/hi/nettrapporter?query=&serie=fisken-og-havet): No. 10/2013, Lunde et al., “Power budget and echo-integrator equations for fish abundance estimation”, and No. 7/2014, Lunde and Korneliussen, “A unifying theory explaining different power budget formulations used in modern scientific echosounders for fish abundance estimation”.

In my opinion, the manuscript should provide more details about how the echo sounder measurements and data processing were conducted. This would support quantitative discussion of measurement uncertainty and possible transferability to other general echo sounder models than those used in the study.

Detail comments

Page 2 line 24: Please check the sentence, “To carry out the quantification of fisheries echo sounders quantitatively …”

Page 4 line 55: The sentence should provide some foundation, e.g., a reference, for the claim that the processes used to survey fishery resources “show no improvement”.

Page 5 line 63: Please consider reformulating the sentence “As the data measured by conventional general echo sounders are not quantifiable, quantifying the data …”. As it stands it seems to imply that the authors will quantify something which is not quantifiable.

Page 7 line 96 and Page 9 line 125: In my opinion, “spherically spreading” or “spherically diverging” would be preferable to “diffuses and attenuates”.

Page 7 line 99: Please clarify the statement “Notably, in quantitative echo sounders, TVG processing can be processed automatically; this is the characteristic difference between quantitative and general echo sounders”. Does this mean, generally, that a general echo sounder does not feature TVG? What implications may this have for the measurement uncertainty, or resolution, of a general echo sounder, when the reception voltage level “digit” is given as an 8-bit integer (page 8 line 107)?

Page 8 line 118: Please clarify the formulation “returned in the source direction by a unit distance”.

Page 8 line 122: Equation (2) does not include the backscattering cross-section of the target. Please change or explain.

Page 8 line 122: Equation (2) is also dimensionally unbalanced. Inclusion of a reference distance R_0 in the spherical spreading and absorption factors would complete it, if G_R is dimensionless.

Page 8 line 123: The formulation “sound pressure of the transmitted wave” seems unclear. Does it refer to the transmitted sound pressure amplitude on the sound beam axis at a reference distance from the source, and, if so, is the target also positioned on the sound beam axis?

Page 8 line 124: Judging by Equation (2), it does not seem correct that the gain G should be in units of dB.

Page 9 line 133: The text should define all the logarithmic quantities in Equation (4), such as making clear that KTR = 10 log_10 K_TR.

Page 9 line 133: From the definition of K_TR above, Equations (4) and (8) seem to define EL as an electrical quantity rather than a sound pressure level as in the classical sonar equation EL = SL – 2TL + TS. The authors refer to Traynor and Ehrenberg, 1990, but I cannot see that they define EL as in the manuscript. The definition of EL must be clarified.

Page 9 line 142: Does Figure 2b exemplify an actual sampling volume? It seems to me that the smallest quantifiable sampling volume would span the whole two-way beam pattern of the transducer, typically illustrated as a conical shell volume corresponding to the two-way equivalent beam solid angle.

Page 10 line 146: Equation (5) does not include the volume backscattering coefficient. Please change or explain.

Page 10 line 147: Consider writing, e.g., “The backscattering volume” or “The ensonified volume of scatterers” rather than “The scattered volume”. Similar formulations are found also elsewhere in the manuscript.

Page 10 line 148: Consider clarifying that psi is the two-way equivalent beam solid angle.

Page 10 line 160: It should be made clear that SV in Equation (9) is not the logarithmic quantity from Equation (8).

Page 11 line 161 and Page 14 line 225: The target strength (TS) is conventionally defined as logarithmic and thus not used as a divisor. Dividing by a mean backscattering cross-section (sigma_bs) can be valid. The authors are encouraged to align their description with the commonly used echo integration formulations as in, e.g., References 27 and 29.

Page 11 line 168: A discussion of the differences between the four measurement frequencies, and possible significance of these differences, would be useful.

Page 11 line 177: It would be useful if Table 1 include the beam angles and two-way beam solid angles of the echo sounders.

Page 12 line 181: Please clarify the formulation “the seabed had not changed significantly”. The formulation is clearer but still somewhat unspecific in the Conclusions, Page 30 line 471.

Page 14 line 225: In my opinion, a more detailed discussion of the secondary validation comparing fish abundance estimated from SA would be useful. Factors such as school size, density, uniformity across the sound beam, edge effects, etc., and how such effects were treated, may influence the measurement results.

Page 15 line 235: Please clarify if by “acoustic index” here it is meant the same as “acoustic indicator” later in the manuscript.

Page 15 line 235: Please clarify the reasoning indicated by sentence “… the seabed has the strongest scatterer in the ocean; therefore, the maximum echo in one ping can be considered the primary reflection …”

Page 15 line 235: How was the maximum echo amplitude from the seafloor calculated? Could there be differences (integration time, sample rate, etc.) between the quantitative and general echo sounders, and between different models of general echo sounders? What importance could this have for the proposed calibration method?

Page 17 line 262: What were the measurement parameters, such as seawater salinity and temperature, transmit power / source level, acoustic beam width, and capability to manoeuvre the calibration ball, and how may they affect the measurement uncertainty and the uncertainty of the theoretical value for the TS of the calibration ball?

Page 18 line 280: It would be useful to include a discussion of what uncertainty and variability should be expected for the four anchovy target strengths, both in the present measurements and in the values from literature. For example, a listing of the 45 extracted SA values could be helpful.

Page 19 line 296: The fish abundance listed in Table 2 for the 120 kHz measurement frequency is only one-third of the results for 38 kHz and 15 kHz, which seem to show excellent agreement. It seems that Table 2 could be discussed in further depth, ideally based on a notion of what measurement uncertainties to expect in general and what is needed for the proposed method to be worthwhile.

Page 19 line 301: Is the mentioned difference of 0.3 dB the same value as used to calibrate (adjust) the values listed in Table 2 as “Cal using calibration ball”?

Page 19 line 303: This passage may be reworked if details of the measurement parameters and their importance are added, see my comment to Page 17 line 262.

Page 19 line 305: Please explain how the 0.5 dB calibration measurement uncertainty was estimated – preferably in the description of the measurements (above). It would be useful to modify the very general claim “the results of this study were very accurate” to refer more specifically to the agreement between measured and theoretical TS of the calibration ball without adjustment of the data from the general echo sounders.

Page 20 line 308: What are the beam angles for the echo sounders used in this study (ref. the comment to Table 1)?

Page 20 line 311: The sentence “Therefore, …” should be more specific in its description of why calibrated general echo sounders can be used as “quasi-quantitative” echo sounders.

Page 20 line 314: The sentence may over-state the accuracy of the general echo sounder, if based only on the calibration ball measurement. Please consider revising the statement, or revealing more details of the measurements to found the claim more firmly.

Page 21 line 322: Please clarify what is means that the “echo number” is 4608. Is it the number of echoes that were acquired and used, and is it the same between the echo sounders?

Page 21 line 322: What is meant by “extracted at one ping”? Does this refer to the statistics of single echoes as opposed to the averages within grid cells discussed later?

Page 21 line 324: Please clarify the formulation “… primary reflections saturated”. Did the seabed echoes saturate the quantitative echo sounders, explaining why these measurements are missing from Table 3? The word “thus” may be somewhat overused.

Page 21 line 328: Please specify that the values in Table 3 are given in decibels, and what reference is used for the dB value.

Page 22 line 334: The minimum SV based on the secondary reflection seem very low. The text could benefit from a discussion of factors that could cause weak secondary echoes compared to the primary echoes (in addition to the brief discussion on grain size on Pages 24-25). Is the mean value based on all the recorded values, and could there be some benefit in introducing a cut-off value or some other means to reduce the influence of weak measured echoes?

Page 22 line 345: Table 4: Same comment as for Table 3. Also, are all results in Table 4 mean values per grid cell, as opposed to single-echo statistics in Table 3?

Page 25 line 374: Please define what is meant by the echo convergence time.

Page 24 line 375: Please reconsider the statement that the acoustic impedance of sand particles is smaller than that of seawater.

Page 25 line 377: Please consider revising the statement that it is the acoustic impedance of gravel that makes the reflection more diffuse. Could it be that the echo from a gravel seabed has more of a scattering-dominated characteristic, due to roughness, rather than specular reflection from a smoother sand or sediment bottom?

Page 26 line 400: Please clarify what is meant by “saturated”.

Page 26 line 402: Please revise the statement “if the discharged sound waves are strong … the sound waves may get reflected before being attenuated”. As explained earlier in the paper, propagating sound waves are always attenuated.

Page 26 line 407: Please revise the statement “Therefore, the backscattering strength tends to be a non-linear phenomenon”. The text leading to the statement does not imply nonlinearity in the process of backscattering but may point to weaknesses in the applicability of the proposed calibration method to the echo sounders used.

Page 27 line 420: What does the achieved agreement mean for possible application of general echo sounders for quantitative survey measurements of fish abundance?

Page 29 line 442: Please clarify the sentence “The seabed data captured …”

Page 29 line 448: In my opinion, the work would benefit from a further discussion of why the adjustments in SV measured by the general echo sounders are so large while the TS of calibration balls were measured with only 0.3 dB difference from theoretical values.

Page 31 line 470: The Conclusions chapter introduces new and important items related to both possible applications, technical aspects of quantitative measurement of the secondary bottom reflection, and the frequency dependence of backscattering strength. These items should be introduced in the appropriate introduction and discussion sections earlier in the text.

Reviewer #2: Summary:

The main content of the manuscript is the development of a simple calibration method using the seabed backscattering strength. This technique offers a solid framework for calibrating echo sounders used in commercial fisheries, enhancing resource estimation and management. The authors tested their metholodogy comparing with echo sounders calibrated using a calibration ball in multiple scenarios.

Major comments:

The manuscript shows a lot of promise, but some major issues need to be addressed before it can be published from my point of view:

• I found the manuscript difficult to read at some points, specially the “Results and discussion” section. Most of the large amount of data they describe in these paragraphs may be tabulated and then only comment the relevant features in the text, thus considerably improving the readibility. Besides that, the authors abuse of the usage of the semicolon at many points, creating very long sentence which are difficult to understand easily. Finally, I command the authors to separate the discussion of Tables 3 and 4 into different paragraphs for more clarity.

• I miss more connection between the “Mechanics of general echo sounders” and the “Results and discussion” sections. The computations that yield the tables should be clearly referenced to facilitate the understanding of non-expert readers.

• All the research performed in the “Results and discussion” section should be accompained by spread measures of the data, i.e., it is not sufficient to provide mean values of the quantities, but also the standard deviations must be detailed.

• I feel that the future work can be extended in some directions. For example, echo sounder are widely used in dFAD fishing, and their calibration is crucial both for fisherman and for research due to the large amount of data they provide. Have you considered to use this new calibration system to the echo sounder equipped in dFADs?

Minor comments:

Abstract

• L22. Change semicolon by full stop.

• L24. I feel “quantification of fisheries echo sounders quantitatively” to be redundant.

• L27. “Therefore” is not necessary here.

• L28. I suggest changing “The method involves using” by “Our approach is based on the usage of”

• L31. The semicolon is not necessary: “...the seabed echoes, which were calibrated by comparing…”

Introduction

• L44. Are being depleted.

• L49. I suggest to substitute “fishes” by “biomass present”.

• L51. I suggest to substitute “quantify” by “estimate”.

• L52. “Several disadvantages”, but only one is mentioned.

• L63-65: This sentence is not clear. Please, clarify.

• L74: I suggest to substitute “Due to” by “For”.

• L76-77. I feel “In this method, we used the backscattering strength of the seabed” to be redundant looking at how the previous sentence ended.

• L80. I suggest to substitute “measure the backscattering strength of the seabed” by “the backscattering strength of the seabed can be measured”.

• L84. What is “inter-ship calibration”? It has not been defined.

Methods

• L91. Change semicolon by full stop.

• L102-103. I suggest to omit the formulas in Fig 1, as they are commented afterwards, so the footnote fully describes the image.

• L106. Change semicolon by full stop.

• L110. The equation in the text are subjected to the same puntuaction rules, so a full stop or a comma (as in this case) must be placed at the end of equation (1).

• L115. What are single echo and group echoes? Please, clarify.

• L115. I suggest to substitute “; therefore, it is” by “, so it is”.

• L121. It find referencing an equation that does not already appear in the text a bit weird. I suggest to substitute “using Eq (2) [17]” by “as” and move the reference [17] after describing the variables.

• L122. The final comma is missing.

• L123-125. I suggest to describe all the parameters involved in Eq (2) just after the equation, and then the authors may point out related observations such as “the factor of transmit and received” and the characteristic of R^{-2}.

• L128. What does “with the characteristic of exp(-2\\alpha R)2 mean?

• L130. Equation (3) is not an equation, as there is no equality. Moreover, the authors must mention in what basis are the logarithms expressed.

• L133. K_{TR} was used in L125, but KTR is used in this line.

• L138-140. P_{0} and R have already been described, and the rest of the parameters are described below. I strongly recommend the authors to better explain the images instead of defining the parameters which are detailed in the text.

• L149. Same comment as in L121.

• L150. The full stop is missing.

• L152. Change semicolon by full stop.

• L153. Again, this is not an equation. Please, describe precisely the relation between the parameters.

• L155. The full stop is missing.

• L156-159. I suggest the authors to motivate better why SA is preferred over SV.

• L158. I suggest to substitute “Notably, SA” by “This quantity is”.

• L160. The R1 and R2 are not defined.

• L161. I suggest to add “in Eq. (4)” after “TS”.

• L161. The number of target species is denoted as “n” in the following section. The authors should consider to include this notation here.

• L166. I do not support the usage of Figure 3, since it does not add any value to the manuscript.

• L167. I suggest removing “in this study” since it is unnecessary here.

• L185. Change semicolon by full stop.

• L185. Add “in this study” after “measurement lines2.

• L185. A linking word is missing before “The depth…”. “Besides that” suits well here.

• L199. There are sentences where low numbers are in letter and others that are in numbers. I suggest taking the same criterion everywhere (preferably, with letter. i.e., “ten” instead of “10”).

• L229. Change semicolon by full stop.

• L236. Change semicolon by full stop.

• L241. I suggest rephasing this sentence as “We treated the record as an error…”

• L242. I suggest rephasing this sentence as “assumed that secondary reflections of the seabed were not captured…”

• L255. The authors of the references were not named until this point (Wentworth). Please, take the same criterion everywhere.

• L256. I suggest changing “based” by “depending”.

Results and discussion

• L265. State the water temperature and salinity.

• L265. A linking word is missing before “There was…”. “Therefore” suits well here.

• L284. The authors of the references were not named until this point (Amakasu et al). Please, take the same criterion everywhere.

• L297. The authors say the differences were low, but there are a 110000 difference between 4th row and 6th row in Table 2, which I think is not negligible.

• L303-306. This four-line sentence is difficult to read. Consider rephrasing it.

• L322. What is the echo number?

• L336. Change semicolon by full stop.

• L360. Replace “10” by “ten”.

• L370. Replace “10” by “ten”.

• L374-375. I do not understand “The difference in acoustic impedance of the medium is large, the reflection is large”.

• L387. What does “acoustic impedance”?

• L403. Change semicolon by full stop.

• L406. I suggest to substitute “; however,” by “, although”.

• L411. Replace “1” by “one”.

• L416. I suggest to move the reference [52] just after “According to a previous study...”.

Conclusions

• L482. Replace “½” by “one-half”.

• L490. Change semicolon by full stop.

**********

6. PLOS authors have the option to publish the peer review history of their article (what does this mean?). If published, this will include your full peer review and any attached files.

If you choose “no”, your identity will remain anonymous but your review may still be made public.

**Do you want your identity to be public for this peer review?** For information about this choice, including consent withdrawal, please see our Privacy Policy.

Reviewer #1: No

Reviewer #2: No

**********

[NOTE: If reviewer comments were submitted as an attachment file, they will be attached to this email and accessible via the submission site. Please log into your account, locate the manuscript record, and check for the action link "View Attachments". If this link does not appear, there are no attachment files.]

While revising your submission, please upload your figure files to the Preflight Analysis and Conversion Engine (PACE) digital diagnostic tool, https://pacev2.apexcovantage.com/. PACE helps ensure that figures meet PLOS requirements. To use PACE, you must first register as a user. Registration is free. Then, login and navigate to the UPLOAD tab, where you will find detailed instructions on how to use the tool. If you encounter any issues or have any questions when using PACE, please email PLOS at figures@plos.org. Please note that Supporting Information files do not need this step.

# Author comment: Author response to Decision Letter 0

**DOI:** 10.1371/journal.pone.0301689.r002

31 Aug 2023

Response to Reviewer 1

We have revised the text according to the comments. And we have added statistical analysis in some parts and clarified definitions of terms. For the data openly available online, since it is part of a national project, data is a bit difficult to make publicly available.

1. Page 2 line 24: Please check the sentence, “To carry out the quantification of fisheries echo sounders quantitatively …

We have changed the sentence to “To carry out the quantification for the fisheries echo sounder, it is important to perform calibration periodically, using a calibration ball”.

2. Page 4 line 55: The sentence should provide some foundation, e.g., a reference, for the claim that the processes used to survey fishery resources “show no improvement”.

The text is a little confusing and has been corrected as follows “Due to these reasons, there has not been much progress in continuous and wide-area fisheries resource surveys using acoustic methods”. And we also added the reference after the sentence.

3. Page 5 line 63: Please consider reformulating the sentence “As the data measured by conventional general echo sounders are not quantifiable, quantifying the data …”. As it stands it seems to imply that the authors will quantify something which is not quantifiable.

We have changed the sentence to “In order to attribute quantifiability to general echo sounders, it is important to compensate for the strength of the reflected sound. Therefore, it is essential to calibrate the transducer system that is processing the sound wave”.

4. Page 7 line 96 and Page 9 line 125: In my opinion, “spherically spreading” or “spherically diverging” would be preferable to “diffuses and attenuates”.

We have changed the “diffuses and attenuates” to the “spherically diverging”.

5. Page 7 line 99: Please clarify the statement “Notably, in quantitative echo sounders, TVG processing can be processed automatically; this is the characteristic difference between quantitative and general echo sounders”. Does this mean, generally, that a general echo sounder does not feature TVG? What implications may this have for the measurement uncertainty, or resolution, of a general echo sounder, when the reception voltage level “digit” is given as an 8-bit integer (page 8 line 107)?

Yes, that means a general echo sounder does not feature TVG in general. Therefore, Therefore, the strength of the received sound (reception voltage level) is not corrected by the TVG process. For example, the seabed is the strongest reflector, and in shallow waters, the reflected sound is very strong. However, the quantitative echo sounder is depth compensated by TVG processing, and the sound is processed by recognizing that it is in a shallow area. Therefore, changes in sound can be seen even in shallow waters. On the other hand, a general echo sounder adds TVG calculations to the received sound, so it returns a strong sound as it is, and no change can be seen in shallow waters. We have added this information in the Results and Discussion Ⅱ section as follows “Unlike quantitative echo sounders, TVG processing cannot be automatically executed as a function in the general echo sounder. Because the TVG calculations were manually added to the recorded dataset, it is impossible to adjust the distance attenuation when recording the data, and the backscattering strength of a strong scatterer like the seabed tends to saturate easily at shallow water depths”.

6. Page 8 line 118: Please clarify the formulation “returned in the source direction by a unit distance”.

For clarity, we have reworded to the following sentence “the power received by an echo-integrator (without TVG) from the distributed target at range R”.

7. Page 8 line 122: Page 8 line 122: Equation (2) does not include the backscattering cross-section of the target. Please change or explain. Equation (2) is also dimensionally unbalanced. Inclusion of a reference distance R_0 in the spherical spreading and absorption factors would complete it, if G_R is dimensionless. Page 8 line 123: The formulation “sound pressure of the transmitted wave” seems unclear. Does it refer to the transmitted sound pressure amplitude on the sound beam axis at a reference distance from the source, and, if so, is the target also positioned on the sound beam axis? Page 8 line 124: Judging by Equation (2), it does not seem correct that the gain G should be in units of dB.

We changed the equation to the “Ets2 = (Po M GR)2 R-4 exp(-4αR) b4 ts”. And we have added the sentence as follows “Where Po (Pa) is the output transmission sound pressure at the transducer terminals amplitude normalized to the 1 m range, M (V/Pa) denotes the receiver’s sensitivity, and GR is the gain of the front-end amplifier. Furthermore, b is the beam pattern (function of direction describing the amplitude sensitivity), and ts is the linear measure of the backscattering cross-section σbs,”.

8. Page 9 line 133: The text should define all the logarithmic quantities in Equation (4), such as making clear that KTR = 10 log_10 K_TR. Page 9 line 133: From the definition of K_TR above, Equations (4) and (8) seem to define EL as an electrical quantity rather than a sound pressure level as in the classical sonar equation EL = SL – 2TL + TS. The authors refer to Traynor and Ehrenberg, 1990, but I cannot see that they define EL as in the manuscript. The definition of EL must be clarified.

We have added the sentence as follows “Where TS is 10 log ts, EL is 10 log Ets, KTR is 10 KTR”.

9. Page 9 line 142: Does Figure 2b exemplify an actual sampling volume? It seems to me that the smallest quantifiable sampling volume would span the whole two-way beam pattern of the transducer, typically illustrated as a conical shell volume corresponding to the two-way equivalent beam solid angle.

No, it is just an illustration of the image. Therefore, to avoid confusion, we have changed the title of Figure 2 to “Explanation of the sonar equation for target strength TS (a) and volume backscattering strength SV (b). TS is the backscattering strength of a single reflector and SV is the backscattering strength of multiple reflectors. Po is the output transmission sound pressure at the transducer terminals; R is the propagation distance; θ is the direction angle; c is the speed of sound; τ is the pulse width; Ψ is the equivalent beamwidth for volume scattering”.

10. Page 10 line 146: Equation (5) does not include the volume backscattering coefficient. Please change or explain. Page 10 line 147: Consider writing, e.g., “The backscattering volume” or “The ensonified volume of scatterers” rather than “The scattered volume”. Similar formulations are found also elsewhere in the manuscript. Page 10 line 148: Consider clarifying that psi is the two-way equivalent beam solid angle.

We changed the equation to the “Esv2 = (Po M GR)2 R-4 exp(-4αR) V sv”. And we have changed the sentence as follows “Where sv is the volume backscattering coefficient. The backscattering volume V, which is the shell thickness multiplied by the effective cross-sectional area of the beam, was calculated from the volume element of thickness cτ/2, using the pulse width, τ, sound speed, c (m/s), and an equivalent beam angle of a transducer, Ψ [22] as shown in Eq. (6)”.

11. Page 10 line 160: It should be made clear that SV in Equation (9) is not the logarithmic quantity from Equation (8).

We changed the equation to the “sa = ∫_R1^R2▒〖sv dR〗”. And we have added the sentence as follows “Where sa is the area backscattering coefficient, SA and SV are the log measure of sa and sv.”.

12. Page 11 line 161 and Page 14 line 225: The target strength (TS) is conventionally defined as logarithmic and thus not used as a divisor. Dividing by a mean backscattering cross-section (sigma_bs) can be valid. The authors are encouraged to align their description with the commonly used echo integration formulations as in, e.g., References 27 and 29.

We have clarified that all calculations use linear measurements and not logarithmic measurements. And we have changed the sentence as follows “By dividing the calculated value by the ts, which is the target strength of one scatterer (a linear measure of σbs), the number of the target species was calculated, and the distribution density per area within a specific sea area was also obtained”. 

13. Page 11 line 168: A discussion of the differences between the four measurement frequencies, and possible significance of these differences, would be useful.

We have added the information in the Conclusions to discuss the four measurement frequencies as follows “However, while the primary frequencies for commonly used quantitative echo sounders are 38 kHz and 120 kHz, which are less error-prone, 15 kHz and 200 kHz are the most used frequencies for some fisheries to search deeper waters and to better understand fish schools. Therefore, when using a quantitative echo sounder to verify the results of a general echo sounder, it is inevitable that there will be a difference in frequency. Since each frequency has different frequency characteristics, it is preferable to use the biomass obtained from backscattering strength when comparing the quantitative and general echo sounders”.

14. Page 11 line 177: It would be useful if Table 1 include the beam angles and two-way beam solid angles of the echo sounders.

We have added the beam angles of the echo sounders in Table 1. We also added the information about the beam angle of the general echo sounder in the Method section as follows “Since the non-split-beam general echo sounder could not know the position of the calibration ball within the beam, the position of the calibration ball was varied within the beam range to find the position of the peak. The beam width at a low frequency (15 kHz) with a wide beam is 32°, and at a high frequency (200 kHz) with a narrow beam is 6°”.

15. Page 12 line 181: Please clarify the formulation “the seabed had not changed significantly”. The formulation is clearer but still somewhat unspecific in the Conclusions, Page 30 line 471.

We have changed the sentence as follows “and the survey focused on the flat seabed, where the sediment and slope had not changed significantly”. And, in the Conclusion, we have changed "the seabed had not changed significantly" to the "sandy area".

16. Page 14 line 225: In my opinion, a more detailed discussion of the secondary validation comparing fish abundance estimated from SA would be useful. Factors such as school size, density, uniformity across the sound beam, edge effects, etc., and how such effects were treated, may influence the measurement results. Page 20 line 311: The sentence “Therefore, …” should be more specific in its description of why calibrated general echo sounders can be used as “quasi-quantitative” echo sounders.

We should have explained more about this part. So, we have added the sentence in the Results and Discussion I as follows “In the study area, we extracted the data for 45 fish groups as the Japanese anchovy groups, and the SA of each fish group differed depending on the frequency even for the same fish school. The SA obtained from the general echo sounder was -27.8 ~ -18.2 dB at 15 kHz and -23.0 ~ -13.2 dB at 200 kHz. For the quantitative echo sounder, the SA was -34.6 ~ -21.5 dB at 38 kHz and -39.5 ~ -23.5 dB at 120 kHz” and “In addition, there was a difference between the SA of the same fish group measured by the calibrated general echo sounder and the quantitative echo sounder. This is thought to be due to the size of each fish school and the non-uniformity of fish density. Since the beam spread differs depending on the frequency used, it is possible that for the same fish school, the cross-sectional area that can be detected differs depending on the frequency, thereby causing the difference in SA. Meanwhile, the difference between two echo sounders in fish abundance throughout the study area estimated using the SAmean was very small at the low frequencies. In general, in the case of the quantitative echo sounders, the frequency often used for resource assessment is 38 kHz, because low frequencies have a wider beam spread and stronger backscattering strength from the fish school than high frequencies. At the low frequency of two echo sounders, even though differences were observed in each fish school, there was no significant difference in the overall number of fish. Therefore, we believed it will be possible to calculate the fish abundance in the sea using the low frequency of the calibrated general echo sounder”.

17. Page 15 line 235: Please clarify if by “acoustic index” here it is meant the same as “acoustic indicator” later in the manuscript. Page 15 line 235: Please clarify the reasoning indicated by the sentence “… the seabed has the strongest scatterer in the ocean; therefore, the maximum echo in one ping can be considered the primary reflection …”. Page 15 line 235: How was the maximum echo amplitude from the seafloor calculated? Could there be differences (integration time, sample rate, etc.) between the quantitative and general echo sounders, and between different models of general echo sounders? What importance could this have for the proposed calibration method?

We have changed the “acoustic index” to the “acoustic indicator”. And we have explained the analysis method about SV in more detail as follows “When the seabed was used in the analysis, determining the seabed line is essential to distinguish between echoes in the water column and seabed echoes. The echoes, which are below the seabed line, were used to calculate the seabed backscattering strength, and the SV was used as an acoustic indicator. Since there are no scatters stronger than the seabed in the ocean, the depth of maximum SV in each ping can be considered as the seabed” and “In an area, even if the seabed sediments are similar, slight changes in the inclination of the vessel can change the backscattering strength of the seabed. Therefore, to obtain a more stable value, it is necessary to average similar seabed echoes over a certain range using echo integration processing. In this study, the ping rates differed depending on the echo sounder used; however, the distances traveled were the same because they were recorded at the same time. Therefore, when calculating SVmean, the horizontal integration range was based on the distance. The vertical integration range was unified to 1 m from the seabed for both echo sounders”.

18. Page 17 line 262: What were the measurement parameters, such as seawater salinity and temperature, transmit power / source level, acoustic beam width, and capability to manoeuvre the calibration ball, and how may they affect the measurement uncertainty and the uncertainty of the theoretical value for the TS of the calibration ball? Page 19 line 303: This passage may be reworked if details of the measurement parameters and their importance are added, see my comment to Page 17 line 262.

We have added more detailed calibration methods to the Method section. The text is as follows “Since it was necessary to place the calibration ball directly under the transducer, the survey was conducted in an inner bay where waves, wind, and currents were few. The mean wind speed on the day of the survey was 1.6 m/s, and the vessel was anchored from the bow and stern to prevent the vessel from being swept away. During calibration, the calibration ball was hung from three points on the vessel's front, starboard, and port sides with fishing rods so that the calibration ball was directly under the transducer” and “Since the non-split-beam general echo sounder could not know the position of the calibration ball within the beam, the position of the calibration ball was varied within the beam range to find the position of the peak. The beam width at a low frequency (15 kHz) with a wide beam is 32°, and at a high frequency (200 kHz) with a narrow beam is 6°. The maximum TS of the calibration ball was checked using a dedicated tool manufactured by Furuno”. We also added some information in the Result section as follows “In this study, the water temperature was 21.4°C, the salinity was 30.4 psu, and a 38.1 mm WC sphere gives a sound speed c of 1520.2 m /s. When the transmit power was 

Attachment Submitted filename: Response to Reviewer 2.docx

# Peer review: Decision Letter 1

**DOI:** 10.1371/journal.pone.0301689.r003

**Authors:** Vitor Hugo Rodrigues Paiva

24 Oct 2023

PONE-D-23-17337R1Calibration of Commercial Fisheries Echo Sounders for the Estimation and Management of Fishery ResourcesPLOS ONE

Dear Dr. Zhu,

Thank you for submitting your manuscript to PLOS ONE. After careful consideration, we feel that it has merit but does not fully meet PLOS ONE’s publication criteria as it currently stands. Therefore, we invite you to submit a revised version of the manuscript that addresses the points raised during the review process.

Please submit your revised manuscript by Dec 08 2023 11:59PM. If you will need more time than this to complete your revisions, please reply to this message or contact the journal office at plosone@plos.org. When you're ready to submit your revision, log on to https://www.editorialmanager.com/pone/ and select the 'Submissions Needing Revision' folder to locate your manuscript file.

Please include the following items when submitting your revised manuscript:

- A rebuttal letter that responds to each point raised by the academic editor and reviewer(s). You should upload this letter as a separate file labeled 'Response to Reviewers'.
- A marked-up copy of your manuscript that highlights changes made to the original version. You should upload this as a separate file labeled 'Revised Manuscript with Track Changes'.
- An unmarked version of your revised paper without tracked changes. You should upload this as a separate file labeled 'Manuscript'.

If you would like to make changes to your financial disclosure, please include your updated statement in your cover letter. Guidelines for resubmitting your figure files are available below the reviewer comments at the end of this letter.

If applicable, we recommend that you deposit your laboratory protocols in protocols.io to enhance the reproducibility of your results. Protocols.io assigns your protocol its own identifier (DOI) so that it can be cited independently in the future. For instructions see: https://journals.plos.org/plosone/s/submission-guidelines#loc-laboratory-protocols. Additionally, PLOS ONE offers an option for publishing peer-reviewed Lab Protocol articles, which describe protocols hosted on protocols.io. Read more information on sharing protocols at https://plos.org/protocols?utm_medium=editorial-email&utm_source=authorletters&utm_campaign=protocols.

We look forward to receiving your revised manuscript.

Kind regards,

Vitor Hugo Rodrigues Paiva, Ph.D.

Academic Editor

PLOS ONE

[Note: HTML markup is below. Please do not edit.]

Reviewers' comments:

Reviewer's Responses to Questions

**Comments to the Author**

1. If the authors have adequately addressed your comments raised in a previous round of review and you feel that this manuscript is now acceptable for publication, you may indicate that here to bypass the “Comments to the Author” section, enter your conflict of interest statement in the “Confidential to Editor” section, and submit your "Accept" recommendation.

Reviewer #1: (No Response)

Reviewer #3: (No Response)

**********

2. Is the manuscript technically sound, and do the data support the conclusions?

The manuscript must describe a technically sound piece of scientific research with data that supports the conclusions. Experiments must have been conducted rigorously, with appropriate controls, replication, and sample sizes. The conclusions must be drawn appropriately based on the data presented. 

Reviewer #1: Yes

Reviewer #3: No

**********

3. Has the statistical analysis been performed appropriately and rigorously? 

Reviewer #1: Yes

Reviewer #3: No

**********

4. Have the authors made all data underlying the findings in their manuscript fully available?

The PLOS Data policy requires authors to make all data underlying the findings described in their manuscript fully available without restriction, with rare exception (please refer to the Data Availability Statement in the manuscript PDF file). The data should be provided as part of the manuscript or its supporting information, or deposited to a public repository. For example, in addition to summary statistics, the data points behind means, medians and variance measures should be available. If there are restrictions on publicly sharing data—e.g. participant privacy or use of data from a third party—those must be specified.

Reviewer #1: No

Reviewer #3: No

**********

5. Is the manuscript presented in an intelligible fashion and written in standard English?

PLOS ONE does not copyedit accepted manuscripts, so the language in submitted articles must be clear, correct, and unambiguous. Any typographical or grammatical errors should be corrected at revision, so please note any specific errors here.

Reviewer #1: Yes

Reviewer #3: No

**********

6. Review Comments to the Author

Please use the space provided to explain your answers to the questions above. You may also include additional comments for the author, including concerns about dual publication, research ethics, or publication ethics. (Please upload your review as an attachment if it exceeds 20,000 characters)

Reviewer #1: The authors have made considerable changes to the manuscript. The changes have been detailed in an intelligible way, but not all comments have been addressed sufficiently. The manuscript still has some shortcomings. My recommendation is that another revision is called for before the manuscript may be accepted.

To Review Question 3, I have not been able to read clearly from the submittal whether all the data will be made publicly available or not.

Major comments

Revision 1 (R1) does not seem to respond to the first two of my original three major comments. These are repeated and amended below. The page and line number references have been updated to match R1.

1. The authors state that “the calibration of general echo sounders has never been carried out using the backscattering strength of the seabed” (page 5 line 85). In my opinion, the manuscript should account for, and compare with, other uses of the seabed as sonar calibration reference, such as, e.g., Eleftherakis et al., Mar. Geophys. Res. 39 (2018), Weber & Ward, J. Acoust. Soc. Am. 138 (2015), Roche et al., Mar. Geophys. Res. 39 (2018). The reviewer found the two latter references via the Master’s theses of Guimarães (2020), and Cândido (2022).

2. The manuscript should define the quantities and equations used more precisely and in line with the well-established literature in the field. Some, but not all, details are noted under “Detail comments” below. A thorough foundation for the equations built upon in the manuscript are given in the report series “Fisken og havet” (The Fish and the Sea), available online at Institute of Marine Research (https://www.hi.no/en/hi/nettrapporter?query=&serie=fisken-og-havet): No. 10/2013, Lunde et al., “Power budget and echo-integrator equations for fish abundance estimation”, and No. 7/2014, Lunde and Korneliussen, “A unifying theory explaining different power budget formulations used in modern scientific echosounders for fish abundance estimation”. I strongly recommend to use the terms and definitions found in these references, or to refer to literature that establishes the particular terms and definitions of the manuscript.

Below I have made some detail comments to the changes made in R1.

Detail comments

Page 6 line 96: An original formulation containing “diffuses and attenuates” was replaced in R1 by “The echoes include distance attenuation, e.g., spherically diverging attenuations caused by the propagation of sound waves”. I propose to rephrase once more, to make it clear that it is not the attenuations that diverge. If the two mechanisms of attenuation considered are absorption and spherical spreading, I propose that these two are mentioned, without “e.g.”.

Page 7 line 121: A series of comments were resolved by adjusting Equation (2) and the section around it. The sentence on Line 121 now includes the terms “single echo”, “single object”, “backscattering strength”, “target strength", “reflector”, and “distribution density”. I propose to rephrase this sentence and possibly divide it into multiple sentences explaining more clearly what Equation (2) describes.

Page 8 line 126: Please clarify what “terminals” the transmission sound pressure P_0 refers to, and what is meant by “normalized to the 1 m range”. Standard wording is found in acoustics textbooks and e.g. the “Fisken og havet” reference mentioned in my second major comment.

Page 8 line 130: Please clarify the definition of “ts” as a “linear measure of” the backscattering cross-section. A standard definition is TS = 10 log_10 (sigma_bs / r_0^2). This or any alternative definition should be clearly defined in mathematical terms.

Page 8 line 133: Please reconsider the description “the sound waves absorb energy from the components of seawater”.

Page 8 line 139: The echo level is usually defined as the free-field sound pressure level due to backscattering, incident at the position of the sonar transducer. The alternative definition given here as a received voltage squared after front-end amplification warrants a further, mathematically stringent, explanation.

Page 10 line 148: Consider clarifying that psi is the two-way equivalent beam solid angle.

Page 11 line 168: A discussion of the differences between the four measurement frequencies, and possible significance of these differences, would be useful. Please clarify what is meant by that “each frequency has different frequency characteristics”, and why this is an argument for using backscattering strength to compare measurements taken at different frequencies.

Page 11 line 196: Thank you for adding beam angles to Table 1. When comparing measurements of the seafloor, are not also the two-way beam solid angles psi needed?

Page 18 line 304: Please rephrase “a 38.1 mm WC sphere gives a sound speed c of 1520.2 m/s”.

Page 29 line 465: Consider inserting “measured”, “observed”, or “apparent” in the sentence – “there was no change in the measured SV values of the seabed primary reflection”.

Page 32 line 524: What does the achieved agreement mean for the achievable measurement accuracy in terms of SV when general echo sounders are used for quantitative survey measurements of fish abundance?

Reviewer #3: From the perspective of this reviewer, while technique of using the seabed for calibration is well established, especially within the hydrographic community, the application to the use of fish finders, as far as this reviewer is aware, is the novelty of this work. However, the manuscript as currently presented does not seem to provide enough evidence to substantiate many of the claims made in the results and the discussion, and the manuscript, overall, lacks the focus that is conveyed in the abstract.

Further, from an acoustics perspective, many of the definitions and processes described in the methods and then used throughout the paper are both inconsistent internally within the manuscript and fail to follow field-specific conventions (e.g., see those presented by MacLennan et al., 2002). In addition to confusing an informed reader, this adds further skepticism to the reported results as some of the important details of the methods and analyses are either unreported or construed. There are many elements to the manuscript that in its current form read as disjointed, and additional analyses, clarity, or figures (detailed below) are needed to convey the findings the authors to conclude.

Specific comments:

Consider a title that notes the use of seabed backscatter

Line 39, specify what is meant by “them”, i.e., change to “resource”

Line 43, “scientifically” is not needed

Line 48, “short time” is unclear, consider rephrasing that they can efficiently estimate biomass over a wide area.

Line 48, Remove “In addition”, not necessary in this statement.

Line 55, 2nd and 3rd commas are not necessary

Line 58, move “to survey resources” to the end of the sentence

Line 67, remove “in the sea”

Paragraph at line 70, really needs to include a lot of references, and discuss what already is/isn’t being done with regards to seabed calibration. There is lot of work in the seafloor mapping field, plus some work with water column fisheries echosounders (See De Robertis and Hjellvic). They need to develop more on why using the seabed works, which currently has 0 references.

Line 94, What is meant by “upgrade”?

Line 92, Replace distance with range throughout this paragraph. What is the ‘essential role’? Clarify what is meant here.

Line 98, TVG does not improve the reception sensitivity, it amplifies the received signal as a function of range. Clarify.

Line 103/Figure 1, r is range. The proper term is SV¸ where the ‘V’ is subscript. See Maclennan et al., 2002 A consistent approach to definitions and symbols in fisheries

acoustics”. Fix throughout.

Line 119, The use of 20log and 40log for volume and individual scattering, respectively, needs to be clarified in the paper. Are you doing both, and if so, provide the equations.

Line 130, σbs is linear, clarify and replace ‘ts’ in equation 2 with σbs. Fix throughout.

Line 139, Again, use σbs in place of ‘ts’, as is consistent with MacLennan et al., 2002.

Line 156/Equation 4, Proper use is subscript of the V, as in sV.

Figure 2, This figure indicates the basic concepts and terminology used in fisheries acoustics and seems to be excessive to include in this manuscript. In addition, these figures already exist in common acoustic literature. This one in particular looks similar to Figure 1 and Figure 2 in Furusawa 2021. Consider removing or citing previous work instead.

Paragraph at 167 and equation 7, The area backscattering coefficient is sa and the units here are incorrect, again see MacLennan et al., 2002.

Line 169, This sentence is unclear. What does layer number have to do with sA? This statement can be removed.

Line 174, R1 and R2 represent the lower and upper ranges of the volume over which sV is being integrated. Clarify this sentence.

Line 175, ‘ts’, as a lower case form, is not a valid representation of the linear form of target strength. Target strength is a logarithmic unit, thus σbs is the linear form. The parenthetical here is incorrect.

Line 176, This is confusing, if you correctly give the units for sa (m2 m-2) then you can clarify that the resulting unit when dividing by σbs (which has units of m2) is target species per unit area.

Figure 3, What are the axes on the seabed backscattering strength maps on the right? Labels are necessary to interpret these. Label the subfigures as (a), (b), and (c) to be able to directly reference them in the text.

Line 189, The figure caption needs to include specific details defining ‘seabed backscattering strength’. Remove the term ‘acoustic indicator’ and refer specifically to SV when discussion volume backscattering strength. The seabed maps look interpolated. Are they? Specify in the caption.

Line 199, Figure 3 does not substantiate the claim that the sediment and slope had not changed significantly, and there is no indication of bathymetry. Consider adding bathymetric contours to the Sv representations in Figure 3.

Line 205, Remove ‘Besides that’

Line 212, What depth was the sphere suspended? Were multiple depths used to confirm the linearity of the response from the general echosounder?

Line 217, Replace ‘know’ with ‘measure’

Line 218, Define ‘peak’ here, which should be referring to the peak backscatter or target strength of the calibration sphere.

Line 218, Remove ‘with a wide beam’ and ‘with a narrow beam’

Line 221, What are the parameters?

Line 225, “to prevent recording the waves reflected from air bubbles”. Does this refer to preventing entrainment of bubbles underneath the transducer? Please clarify.

Line 229, Include sites in the map on figure 1.

Line 235, Did you use a single observation of the maximum TS observed or a mean of an ensemble of observations? Report the number of TS measurements used to calculate the on-axis measurement.

Line 241, Please elaborate what is meant by this sentence.

Line 243, 250, It is unclear how these different calibrations were applied in the processing. Were the echosounder observations corrected afterwards using an offset? Or were data reprocessed using a modified gain based on the observed target strength/seabed measurement? Please expand.

Line 252/Figure 4, Please include at least the depth/range axis and labels on the echograms. Identify the yellow line of the quantitative echogram in the caption. Analysis grid is not defined anywhere in the text. Further, the use of ‘analysis range’ throughout the text is unclear, given the meaning of ‘range’ for volume integration as explained in the methods. Use a different term throughout the text that indicates that this is the horizontal grid.

Line 257, The company name for Echoview is currently Echoview Software Pty Ltd.

Line 258, ‘ts’ should be σbs. This sentence is redundant with previous methods, simply state that fish abundance was calculated for fish schools based on integration of volume backscatter as previously described.

Line 261, Define reaction size and school reactions.

Line 266, ‘treated as fish schools’? Simply state were not included in the analysis.

Line 268, ‘Echoes below the seabed line’ is unclear. Are you referring to the integration volume immediately below the sounder-detected seabed? How was the seabed line detected?

Line 277-280, These two things do not seem to be related as written. Yes, the pitch/roll of the vessel has a significant impact on the observation of the seabed as a result of changes in the incident angle with the seafloor. Was range then used as an indicator of angle? I.e., were only echoes where the seafloor was a consistent range window averaged, assuming that the observations represented the same vessel position? This is highly sensitive to vessel movement and complete flat seabed structure. Were measurements of pitch and roll available to validate this?

Line 282, Clarify that this was 1-m below the detected seabed.

Line 285, This needs to be defined earlier as it has already been used multiple times throughout the text and figures.

Line 286, Earlier it is stated this was done in 50 m grid. Please clarify in either place. Also, due to the confusion between terms, is this the power average (average of the linear values, sV, then converted back into log space)?

Line 290, This information needs to be included in the caption for Figure 1, or Figure 1 should be divided into two figures which can be used in the into/methods (the transect map) and the results (the seabed maps).

Line 294, This is the first time sediments are mentioned. This needs to be described earlier in the survey methods, including how the sediments were collected, and here, additional details into the grain size methods should be included. Define the grain sizes used to distinguish the 3 classes.

Line 306, Again, include the complete unit for TS here, which is dB re 1 m2

Line 304-313 As in the methods, clarify if these are single observations of the maximum TS or an average of the highest values, and if so how many observations were included.

Line 322, use the correct notation, sA, throughout

Line 324-330, sA is not a log unit as being reported. Are these values referring to SV?

Line 332/Table 2, Here as well, these cannot be measurements of sA or any areal unit as they are being reported in decibels. Please correct or clarify.

Line 338-346, Shouldn’t these estimates be roughly the same at both frequencies for the quantitative echosounder?

This indicates that potentially an incorrect target strength values was used. Are these values from the integration using the sphere calibration or the seabed calibration?

Line 354, That is a large range with significant implications for the target strength, and it is assumed that the sound speed did not change over the course of the survey. The difference in TS can be driven by many other factors that are internal to the processing of the echosounder. Please discuss here.

Line 360-372, There is currently very little evidence for the claims described in this paragraph outside of the single aggregation of reported values in the paragraph at 339. This needs to be revised or school-by-school results for animal densities need to be included which will remove the variability of comparing different frequencies, from which statistics can be drawn which can include possible error due to both the calibration and target strength values at the differing frequencies.

Line 377, The difference between -168 and -15 dB is still a ~40% difference in backscattering strength. This is considered not significant?

Line 386/Table 3, include the number of observations in the table.

Line 392, I would argue that they did (-25 vs -16.8 is a massive difference in the secondary reflection). Clarify.

Line 399/Figure 5, It has yet to be stated what the intention behind including the data on the secondary reflections is. Why are they included, and what the meaning behind their measurements? Why are you not showing the same figure for the primary bottom, which is a significantly stronger interface and thus the one that should be used for the calibration?

Line 405/Table 4, Shouldn’t the values for the 50 m grid match shoes reported in Table 3? Why are they not? It is unclear what the difference behind the data being presented are.

Line 414, Define saturated here. If the values were saturated, how are the values being presented in the tables variable?

Line 419, What is the implication of the choice of grid size? Expand.

Line 424, In the methods, only 3 groups are mentioned. Please clarify either here or in the methods.

Line 435, The table does not do a great job demonstrating this. A figure, or including mean Sv on the ternary as a color for each site would be very useful.

Line 441, Table 5 does not substantiate this claim.

Line 448, The convergence time of the echo is not previously described or any results shown.

Line 464, Again, what does this refer to? If the values were ‘saturated’, wouldn’t that mean they would be a maximum value? Are you referring to some sort of color or integration threshold? If something is being saturated, how are you collecting measurements?

Line 475-479, This needs to be discussed much earlier in the manuscript to justify the focus of the results on the use of the secondary bottom. It should be explained both in the intro and then reiterated in the methods.

Line 484, This, like the previous statement regarding incidence, assumes a lot about the ability of the boat to remain perfectly level and the seafloor to be uniformly flat. Given that it is unlikely that both of things are perfectly true, you likely need to do more to address variability due to the pitch, roll of the vessel. Given the beam angles, minor changes (fractions of degrees) can lead to a lot of variance. There is likely also a tradeoff, as gridding over a larger area means that the likelihood that the seafloor varies is greater as well.

Line 503, So was the same data used to calculate the average as is being presented to indicate the variability in Sv? This is very confusing.

Line 509, From the abstract, I expected this to be the focus of the manuscript, however the results and discussion of this intercalibration and the integration of the anchovy schools is buried to the end of the paper. Reporting Sv across different frequencies is meaningless for a reader to interpret, and instead should focus on the estimated fish density as reported earlier. Rather than using the entire school, layers/intervals within the school can be used to increase the sample number in addition to the dual-frequency estimates of each system. Further, the sphere itself can be integrated to do a similar comparison.

Line 528, This is the first place that the objective of the manuscript is stated and references the secondary seabed. This needs to be stated earlier, and the justification for not using the primary seabed needs to be expanded.

Line 540, But the gravel areas and sandy areas show roughly the same variability reported in your results?

Line 544, This is not discussed enough earlier, see previous comments regarding roll.

Line 547, Isn’t that the purpose of this study?

Line 557, This contradicts the previous paragraph stating that specific requirements need to be met.

Line 559, What is meant by error prone? These are not the reasons these have become industry standards. The second clause of this sentence needs a reference, 15 kHz is not as common as 18 kHz.

Line 567, This is too general of a statement.

Line 568, ‘eco sounders’ should be echosounders. What is meant by ‘automatically’? Expand.

Line 567, What is a survey system? Survey of what? Be specific. Clarify or remove as this is better explained in the following sentences.

References

Dalen, J., and Lovik, A. 1981. The influence of wind‐induced bubbles on echo integration surveys. Journal of the Acoustical Society of America, 69: 1653–1659

MacLennan, D. N., Fernandes, P. G., and Dalen, J. 2002. A consistent approach to definitions and symbols in fisheries acoustics. ICES Journal of Marine Science, 59: 365-369.

Hjellvik V., De Robertis A.Vessel comparison on the seabed echo: influence of vessel attitude, 2007US Department of Commerce, NOAA Technical Memorandum NMFS-AFSC-171 pg. 34

Alex De Robertis, Vidar Hjellvik, Neal J. Williamson, Christopher D. Wilson, Silent ships do not always encounter more fish: comparison of acoustic backscatter recorded by a noise-reduced and a conventional research vessel, ICES Journal of Marine Science, Volume 65, Issue 4, May 2008, Pages 623–635, https://doi.org/10.1093/icesjms/fsn025

Furusawa, Masahiko (2021) "Volume Scattering and Echo Integration in Fisheries Acoustics Revisited," Journal of Marine Science and Technology: Vol. 29: Iss. 2, Article 1.

**********

7. PLOS authors have the option to publish the peer review history of their article (what does this mean?). If published, this will include your full peer review and any attached files.

If you choose “no”, your identity will remain anonymous but your review may still be made public.

**Do you want your identity to be public for this peer review?** For information about this choice, including consent withdrawal, please see our Privacy Policy.

Reviewer #1: No

Reviewer #3: No

**********

[NOTE: If reviewer comments were submitted as an attachment file, they will be attached to this email and accessible via the submission site. Please log into your account, locate the manuscript record, and check for the action link "View Attachments". If this link does not appear, there are no attachment files.]

While revising your submission, please upload your figure files to the Preflight Analysis and Conversion Engine (PACE) digital diagnostic tool, https://pacev2.apexcovantage.com/. PACE helps ensure that figures meet PLOS requirements. To use PACE, you must first register as a user. Registration is free. Then, login and navigate to the UPLOAD tab, where you will find detailed instructions on how to use the tool. If you encounter any issues or have any questions when using PACE, please email PLOS at figures@plos.org. Please note that Supporting Information files do not need this step.

# Author comment: Author response to Decision Letter 1

**DOI:** 10.1371/journal.pone.0301689.r004

8 Jan 2024

Could you please check the attached file named Response to Reviewer 1 and Response to Reviewer 3?

Attachment Submitted filename: Response to Reviewer 3.docx

# Peer review: Decision Letter 2

**DOI:** 10.1371/journal.pone.0301689.r005

**Authors:** Vitor Hugo Rodrigues Paiva

10 Mar 2024

PONE-D-23-17337R2Calibration of Commercial Fisheries Echo Sounders Using Seabed Backscatter for the Estimation of Fishery ResourcesPLOS ONE

Dear Dr. Zhu,

Thank you for submitting your manuscript to PLOS ONE. After careful consideration, we feel that it has merit but does not fully meet PLOS ONE’s publication criteria as it currently stands. Therefore, we invite you to submit a revised version of the manuscript that addresses the points raised during the review process.

Please submit your revised manuscript by Apr 24 2024 11:59PM. If you will need more time than this to complete your revisions, please reply to this message or contact the journal office at plosone@plos.org. When you're ready to submit your revision, log on to https://www.editorialmanager.com/pone/ and select the 'Submissions Needing Revision' folder to locate your manuscript file.

Please include the following items when submitting your revised manuscript:

- A rebuttal letter that responds to each point raised by the academic editor and reviewer(s). You should upload this letter as a separate file labeled 'Response to Reviewers'.
- A marked-up copy of your manuscript that highlights changes made to the original version. You should upload this as a separate file labeled 'Revised Manuscript with Track Changes'.
- An unmarked version of your revised paper without tracked changes. You should upload this as a separate file labeled 'Manuscript'.

If you would like to make changes to your financial disclosure, please include your updated statement in your cover letter. Guidelines for resubmitting your figure files are available below the reviewer comments at the end of this letter.

If applicable, we recommend that you deposit your laboratory protocols in protocols.io to enhance the reproducibility of your results. Protocols.io assigns your protocol its own identifier (DOI) so that it can be cited independently in the future. For instructions see: https://journals.plos.org/plosone/s/submission-guidelines#loc-laboratory-protocols. Additionally, PLOS ONE offers an option for publishing peer-reviewed Lab Protocol articles, which describe protocols hosted on protocols.io. Read more information on sharing protocols at https://plos.org/protocols?utm_medium=editorial-email&utm_source=authorletters&utm_campaign=protocols.

We look forward to receiving your revised manuscript.

Kind regards,

Vitor Hugo Rodrigues Paiva, Ph.D.

Academic Editor

PLOS ONE

Journal Requirements:

Please review your reference list to ensure that it is complete and correct. If you have cited papers that have been retracted, please include the rationale for doing so in the manuscript text, or remove these references and replace them with relevant current references. Any changes to the reference list should be mentioned in the rebuttal letter that accompanies your revised manuscript. If you need to cite a retracted article, indicate the article’s retracted status in the References list and also include a citation and full reference for the retraction notice.

[Note: HTML markup is below. Please do not edit.]

Reviewers' comments:

Reviewer's Responses to Questions

**Comments to the Author**

1. If the authors have adequately addressed your comments raised in a previous round of review and you feel that this manuscript is now acceptable for publication, you may indicate that here to bypass the “Comments to the Author” section, enter your conflict of interest statement in the “Confidential to Editor” section, and submit your "Accept" recommendation.

Reviewer #1: (No Response)

**********

2. Is the manuscript technically sound, and do the data support the conclusions?

The manuscript must describe a technically sound piece of scientific research with data that supports the conclusions. Experiments must have been conducted rigorously, with appropriate controls, replication, and sample sizes. The conclusions must be drawn appropriately based on the data presented. 

Reviewer #1: Yes

**********

3. Has the statistical analysis been performed appropriately and rigorously? 

Reviewer #1: I Don't Know

**********

4. Have the authors made all data underlying the findings in their manuscript fully available?

The PLOS Data policy requires authors to make all data underlying the findings described in their manuscript fully available without restriction, with rare exception (please refer to the Data Availability Statement in the manuscript PDF file). The data should be provided as part of the manuscript or its supporting information, or deposited to a public repository. For example, in addition to summary statistics, the data points behind means, medians and variance measures should be available. If there are restrictions on publicly sharing data—e.g. participant privacy or use of data from a third party—those must be specified.

Reviewer #1: Yes

**********

5. Is the manuscript presented in an intelligible fashion and written in standard English?

PLOS ONE does not copyedit accepted manuscripts, so the language in submitted articles must be clear, correct, and unambiguous. Any typographical or grammatical errors should be corrected at revision, so please note any specific errors here.

Reviewer #1: Yes

**********

6. Review Comments to the Author

Please use the space provided to explain your answers to the questions above. You may also include additional comments for the author, including concerns about dual publication, research ethics, or publication ethics. (Please upload your review as an attachment if it exceeds 20,000 characters)

Reviewer #1: Page 2 (abstract), Line 23 and Page 4, Line 55: Please clarify “at a high frequency” – that the acoustic frequency is high or that data are gathered often?

Page 2 (abstract), Line 29: Please clarify “averaged over a certain range” – does this mean over a certain area?

Page 5 Line 81: Please check the sentence: Is it the measurements using a calibrated general echosounder that will be used as a standard value for the seabed in that area?

Page 5 Line 83: I advise to specify clearer what “the validation opportunity” could be, e.g., when the fishing vessel has opportunity to pass over an area where a reference value has been established.

Page 6 Line 93: Consider rephrasing the sentence for conciseness. What is it to “calculate an echo”, and what is it to “quantify the data”?

Page 6 line 96, Page 8 line 101: The term “spherical spreading” is preferable over “diffuse attenuation”.

Page 6 line 99: If the absorption law is exp(-2\\alpha R), the unit of \\alpha should be Np/m not dB/m.

Page 6 Line 99: Consider removing “Therefore, “. Alternatively, remove the whole sentence since the purpose of TVG was already stated in Line 94.

Page 6 Line 101, Page 15 Line 259, Page 24 Line 383: It is stated in the first location that some recent general fisheries echosounders include TVG. What is the difference between such TVG and what is here referred to as “automatic TVG”? Since this is emphasized as a characteristic difference between quantitative and general echosounders I advise to describe the difference in more length. On Line 259 it is stated that general echosounders do not include TVG, while a different formulation again is used on line 383. Please check agreement throughout the text. Also consider to reduce the number of times this is mentioned.

Page 7 line 122, Page 8 line 130: Please check the sentence, it seems to imply that an area is being backscattered.

Page 8 line 136: It may be useful to define two absorption coefficients, or alternatively use 10^(-\\alpha R/20) as the amplitude absorption factor throughout. The units of \\alpha in this line is dB/m, while the \\alpha in Equation (2) is in Np/m if exp means the natural exponential function.

Page 8 line 139: Please check if a “log” is missing from “KTR is 10 K_TR.”

Page 8 line 139: TS is 10 log (\\sigma_bs / r_0^2). Similarly for definitions of logarithmic quantities throughout, please check if reference quantities are missing.

Page 9 line 151: Please check the sentence. Can it be divided?

Page 10-11 table 1: It would be helpful for the reader with a brief explanation already here about of these high transmit powers were chosen. Korneliussen et al., ICES Journal of Marine Science, 65(6), 2008, contains recommendations that could be relevant for comparison.

Is the “Beam angle” in the table equal to what is referred to as the beam width in the text? (Often, the beam width is understood as two times the -3 dB beam angle.)

Page 13 line 223: It is stated that direct comparison between measured target strengths between the general and scientific echosounders is difficult because the measurement frequencies are different. A verification is therefore made by comparing the measurement results in terms of fish abundance. Is this the whole motivation for measuring on fish in the present work? The text should explain clearly how this approach overcomes the problem that the frequencies are different, and why the measurements on fish are desirable in addition to measurements on the calibration ball.

Page 14 line 246: Please review the sentence “When comparing …”, which is difficult to follow.

Page 18 line 290: Please adjust to clarify the calibration state of the echosounder for this first measurement (and for the other results reported). Was it made before any calibration attempts, with factory adjustment values, or after some other form of calibration? Was the initial KTR of the 15 kHz echosounder different from the new value reported at Line 302?

Page 21 line 346: Something seems to be missing from this sentence.

Page 22 line 349: Is this section heading meant to encompass all the text until the next numbered “Results and discussion” heading? It appears that not all of it focuses on “1 ping” measurements. For example, consider adjusting the heading text.

Page 24 line 392: Consider to revise the statement “… echo sounders were found to be larger … ”

Page 27 line 432: This section should discuss why the mean value of S_Vmean increases so strongly with the grid size.

Page 27 line 433: It would be helpful with a discussion of why the maximum values are the same for all the grid sizes.

Page 31 line 490: This still seems unclear. I have not been able to read from the text exactly what signals saturate the receiver. With “saturation of the fish backscattering strength”, is it meant that echoes from fish saturate the receiver? If so, the text would benefit from a discussion of how this leads to an overestimate of abundance.

Page 31 line 492: If the two calibration methods lead to the same abundance measurement results, were the calibration factors almost equal and this result already expected?

Page 32 line 507: Please revise to clarify what is meant by that the slope of the seabed was not smaller than one-half of the beam width. For example, is the slope the angle between the seabed plane and the horizontal? Perhaps a brief definition of slope could be included where the word is first used.

Page 33 line 515: Consider removing the word “While”.

Page 33: With regard to possible reasons for the observed variations, did you also consider the variability in the first two reflections (bottom and surface) affect the sound beam and thus e.g. the footprint (backscattering area) contributing to the second bottom reflection? How were the weather conditions, with respect to scattering at the surface? Alternatively, would it be possible to reduce the transmit power while calibrating, to be able to use the first bottom echo, combined with a study of the uncertainty resulting from changing the transmit power between high and low? This would of course increase the personnel workload somewhat.

**********

7. PLOS authors have the option to publish the peer review history of their article (what does this mean?). If published, this will include your full peer review and any attached files.

If you choose “no”, your identity will remain anonymous but your review may still be made public.

**Do you want your identity to be public for this peer review?** For information about this choice, including consent withdrawal, please see our Privacy Policy.

Reviewer #1: No

**********

[NOTE: If reviewer comments were submitted as an attachment file, they will be attached to this email and accessible via the submission site. Please log into your account, locate the manuscript record, and check for the action link "View Attachments". If this link does not appear, there are no attachment files.]

While revising your submission, please upload your figure files to the Preflight Analysis and Conversion Engine (PACE) digital diagnostic tool, https://pacev2.apexcovantage.com/. PACE helps ensure that figures meet PLOS requirements. To use PACE, you must first register as a user. Registration is free. Then, login and navigate to the UPLOAD tab, where you will find detailed instructions on how to use the tool. If you encounter any issues or have any questions when using PACE, please email PLOS at figures@plos.org. Please note that Supporting Information files do not need this step.

# Author comment: Author response to Decision Letter 2

**DOI:** 10.1371/journal.pone.0301689.r006

19 Mar 2024

1. Page 2 (abstract), Line 23 and Page 4, Line 55: Please clarify “at a high frequency” – that the acoustic frequency is high or that data are gathered often? Page 2 (abstract), Line 29: Please clarify “averaged over a certain range” – does this mean over a certain area?

We have made the changes as commented.

2. Page 5 Line 81: Please check the sentence: Is it the measurements using a calibrated general echosounder that will be used as a standard value for the seabed in that area?

That is correct. We have therefore amended the document to read: The standard value for calibration using the seabed is the backscattering strength in a given area measured with a calibrated general echo sounder.

3. Page 5 Line 83: I advise to specify clearer what “the validation opportunity” could be, e.g., when the fishing vessel has opportunity to pass over an area where a reference value has been established.

We have made the changes as commented.

4. Page 6 Line 93: Consider rephrasing the sentence for conciseness. What is it to “calculate an echo”, and what is it to “quantify the data”?

We have changed the text as follows: Therefore, the backscattering strength of the object targets can be calculated from the echoes obtained by a general echo sounder.

5. Page 6 line 96, Page 8 line 101: The term “spherical spreading” is preferable over “diffuse attenuation”. Page 6 line 99: If the absorption law is exp(-2αR), the unit of α should be Np/m not dB/m. Page 6 Line 99: Consider removing “Therefore, “. Alternatively, remove the whole sentence since the purpose of TVG was already stated in Line 94.

We have made the changes as commented.

6. Page 6 Line 101, Page 15 Line 259, Page 24 Line 383: It is stated in the first location that some recent general fisheries echosounders include TVG. What is the difference between such TVG and what is here referred to as “automatic TVG”? Since this is emphasized as a characteristic difference between quantitative and general echosounders I advise to describe the difference in more length. On Line 259 it is stated that general echosounders do not include TVG, while a different formulation again is used on line 383. Please check agreement throughout the text. Also consider to reduce the number of times this is mentioned.

We have added the following documents: Unlike the general echo sounder, the TVG function is included in the main unit of the quantitative echo sounder. Therefore, the echoes obtained from the quantitative echo sounder are the values after the TVG calculation. And we also checked the agreement throughout the text.

7. Page 7 line 122, Page 8 line 130: Please check the sentence, it seems to imply that an area is being backscattered.

We have changed the sentences to Target strength TS (dB re 1 m2, hereinafter referred to as dB) is a logarithmic measure of the ts, which is the proportion of the incident energy that is backscattered by a single target. The ts is normally described using the backscattering cross-section σbs (m2) and distance R (m) from the target.

8. Page 8 line 136: It may be useful to define two absorption coefficients, or alternatively use 10^(-\\alpha R/20) as the amplitude absorption factor throughout. The units of \\alpha in this line is dB/m, while the \\alpha in Equation (2) is in Np/m if exp means the natural exponential function.

We have used 10(-αR/10) as the amplitude absorption factor throughout.

9. Page 8 line 139: Please check if a “log” is missing from “KTR is 10 K_TR.” Page 8 line 139: TS is 10 log (\\sigma_bs / r_0^2). Similarly for definitions of logarithmic quantities throughout, please check if reference quantities are missing. Page 9 line 151: Please check the sentence. Can it be divided? Page 14 line 246: Please review the sentence “When comparing …”, which is difficult to follow.

We have made the changes as commented.

10. Page 10-11 table 1: It would be helpful for the reader with a brief explanation already here about of these high transmit powers were chosen. Korneliussen et al., ICES Journal of Marine Science, 65(6), 2008, contains recommendations that could be relevant for comparison.

Is the “Beam angle” in the table equal to what is referred to as the beam width in the text? (Often, the beam width is understood as two times the -3 dB beam angle.)

We have changed the beam angle to the -3 dB beam width in Table 1. In addition, we have added the information about transmit power as follows: Note that the general echo sounder, which is a single beam, was set to a strong transmit power even at high frequency to capture fish school responses in deeper waters. In the case of the general echo sounder, which is a split beam, the transmit power at a higher frequency was lower than at a lower frequency to avoid nonlinear effects.

11. Page 13 line 223: It is stated that direct comparison between measured target strengths between the general and scientific echosounders is difficult because the measurement frequencies are different. A verification is therefore made by comparing the measurement results in terms of fish abundance. Is this the whole motivation for measuring on fish in the present work? The text should explain clearly how this approach overcomes the problem that the frequencies are different, and why the measurements on fish are desirable in addition to measurements on the calibration ball.

We have changed the text as follows: In addition, general echo sounders will be used for stock estimation in the future. Therefore, we conducted a secondary validation by comparing the fish abundance measured from the calibrated general and quantitative echo sounders. However, since the frequencies of the general and the quantitative echo sounders used in this study are different, it is difficult to make a comparison using the backscattering strength of the fish school directly. Therefore, we verified the accuracy by comparing the fish numbers calculated using Eq. (7) from both echo sounders. 

12. Page 18 line 290: Please adjust to clarify the calibration state of the echosounder for this first measurement (and for the other results reported). Was it made before any calibration attempts, with factory adjustment values, or after some other form of calibration? Was the initial KTR of the 15 kHz echosounder different from the new value reported at Line 302? Page 24 line 392: Consider to revise the statement “… echo sounders were found to be larger … ”.

This section is a little confusing and has been removed.

13. Page 21 line 346: Something seems to be missing from this sentence.

We have changed the sentence to: The use of too high a power level for the transmission of sound leads to a significant generation of sound at higher frequencies [Reference]. Therefore, resource estimation in shallow water using the high frequency of general echo sounders is considered necessary to adjust electrical transmit power to the extent that it does not interfere with fishing operations.

Korneliussen RJ, Diner N, Ona E, Berger L, Fernandes PG. Proposals for the collection of multifrequency acoustic data. ICES Journal of Marine Science. 2008; 65: 982–994. doi: 10.1093/icesjms/fsn052.

14. Page 22 line 349: Is this section heading meant to encompass all the text until the next numbered “Results and discussion” heading? It appears that not all of it focuses on “1 ping” measurements. For example, consider adjusting the heading text. 

We have made the changes as commented.

15. Page 27 line 432: This section should discuss why the mean value of S_Vmean increases so strongly with the grid size. Page 27 line 433: It would be helpful with a discussion of why the maximum values are the same for all the grid sizes.

Since the values in the table include outliers, table 5 has been reworked. We also have included in the document a discussion of why the Sv values increase strongly with the grid size.

16. Page 31 line 490: This still seems unclear. I have not been able to read from the text exactly what signals saturate the receiver. With “saturation of the fish backscattering strength”, is it meant that echoes from fish saturate the receiver? If so, the text would benefit from a discussion of how this leads to an overestimate of abundance.

We have changed the text to However, as discussed in the previous section, the high-frequency transmission power used in this study was made stronger to search for fish in deeper waters. Therefore, calibration performed by two methods may not correctly correct for backscattering strength, resulting in a strong reflection of shallow water and an overestimation of the resource.

17. Page 31 line 492: If the two calibration methods lead to the same abundance measurement results, were the calibration factors almost equal and this result already expected?

Yes, it is. There was no significant difference in the calibration factors. However, since this study will be used for resource estimation in the future, we think it would be easier to show the difference between the two in terms of fish abundance.

18. Page 32 line 507: Please revise to clarify what is meant by that the slope of the seabed was not smaller than one-half of the beam width. For example, is the slope the angle between the seabed plane and the horizontal? Perhaps a brief definition of slope could be included where the word is first used. Page 33 line 515: Consider removing the word “While”.

We have made the changes as commented.

Page 33: With regard to possible reasons for the observed variations, did you also consider the variability in the first two reflections (bottom and surface) affect the sound beam and thus e.g. the footprint (backscattering area) contributing to the second bottom reflection? How were the weather conditions, with respect to scattering at the surface? Alternatively, would it be possible to reduce the transmit power while calibrating, to be able to use the first bottom echo, combined with a study of the uncertainty resulting from changing the transmit power between high and low? This would of course increase the personnel workload somewhat.

We also considered the impact of the bottom and the surface. It is quite difficult to discuss this part in this study. Therefore, as a future step, we plan to conduct the same survey in various weather conditions to see how much they affect the calibration. In addition, it is impossible to adjust the transmit power easily. Therefore, another future task is to find a transmit power that is balanced between operation and survey. In the future, we plan to increase the accuracy by conducting more studies.

Attachment Submitted filename: Response to Reviewer 1.docx

# Editor report: Decision Letter 3

**DOI:** 10.1371/journal.pone.0301689.r007

**Authors:** Vitor Hugo Rodrigues Paiva

20 Mar 2024

Calibration of Commercial Fisheries Echo Sounders Using Seabed Backscatter for the Estimation of Fishery Resources

PONE-D-23-17337R3

Dear Dr. Zhu,

We’re pleased to inform you that your manuscript has been judged scientifically suitable for publication and will be formally accepted for publication once it meets all outstanding technical requirements.

Within one week, you’ll receive an e-mail detailing the required amendments. When these have been addressed, you’ll receive a formal acceptance letter and your manuscript will be scheduled for publication.

An invoice will be generated when your article is formally accepted. Please note, if your institution has a publishing partnership with PLOS and your article meets the relevant criteria, all or part of your publication costs will be covered. Please make sure your user information is up-to-date by logging into Editorial Manager at Editorial Manager® and clicking the ‘Update My Information' link at the top of the page. If you have any questions relating to publication charges, please contact our Author Billing department directly at authorbilling@plos.org.

If your institution or institutions have a press office, please notify them about your upcoming paper to help maximize its impact. If they’ll be preparing press materials, please inform our press team as soon as possible -- no later than 48 hours after receiving the formal acceptance. Your manuscript will remain under strict press embargo until 2 pm Eastern Time on the date of publication. For more information, please contact onepress@plos.org.

Kind regards,

Vitor Hugo Rodrigues Paiva, Ph.D.

Academic Editor

PLOS ONE

Additional Editor Comments (optional):

Reviewers' comments:

# Editor report: Acceptance letter

**DOI:** 10.1371/journal.pone.0301689.r008

**Authors:** Vitor Hugo Rodrigues Paiva

26 Apr 2024

PONE-D-23-17337R3 

PLOS ONE

Dear Dr. Zhu, 

I'm pleased to inform you that your manuscript has been deemed suitable for publication in PLOS ONE. Congratulations! Your manuscript is now being handed over to our production team.

At this stage, our production department will prepare your paper for publication. This includes ensuring the following:

* All references, tables, and figures are properly cited

* All relevant supporting information is included in the manuscript submission,

* There are no issues that prevent the paper from being properly typeset

If revisions are needed, the production department will contact you directly to resolve them. If no revisions are needed, you will receive an email when the publication date has been set. At this time, we do not offer pre-publication proofs to authors during production of the accepted work. Please keep in mind that we are working through a large volume of accepted articles, so please give us a few weeks to review your paper and let you know the next and final steps. 

Lastly, if your institution or institutions have a press office, please let them know about your upcoming paper now to help maximize its impact. If they'll be preparing press materials, please inform our press team within the next 48 hours. Your manuscript will remain under strict press embargo until 2 pm Eastern Time on the date of publication. For more information, please contact onepress@plos.org.

If we can help with anything else, please email us at customercare@plos.org.

Thank you for submitting your work to PLOS ONE and supporting open access. 

Kind regards, 

PLOS ONE Editorial Office Staff

on behalf of

Dr. Vitor Hugo Rodrigues Paiva 

Academic Editor

PLOS ONE
